# Random generalized linear model: a highly accurate and interpretable ensemble predictor

**DOI:** 10.1186/1471-2105-14-5

**Published:** 2013-01-16

**Authors:** Lin Song, Peter Langfelder, Steve Horvath

**Affiliations:** 1 Human Genetics, David Geffen School of Medicine, University of California, Los Angeles, California, USA; 2 Biostatistics, School of Public Health, University of California, Los Angeles, California, USA

## Abstract

**Background:**

Ensemble predictors such as the random forest are known to have superior accuracy but their black-box predictions are difficult to interpret. In contrast, a generalized linear model (GLM) is very interpretable especially when forward feature selection is used to construct the model. However, forward feature selection tends to overfit the data and leads to low predictive accuracy. Therefore, it remains an important research goal to combine the advantages of ensemble predictors (high accuracy) with the advantages of forward regression modeling (interpretability). To address this goal several articles have explored GLM based ensemble predictors. Since limited evaluations suggested that these ensemble predictors were less accurate than alternative predictors, they have found little attention in the literature.

**Results:**

Comprehensive evaluations involving hundreds of genomic data sets, the UCI machine learning benchmark data, and simulations are used to give GLM based ensemble predictors a new and careful look. A novel bootstrap aggregated (bagged) GLM predictor that incorporates several elements of randomness and instability (random subspace method, optional interaction terms, forward variable selection) often outperforms a host of alternative prediction methods including random forests and penalized regression models (ridge regression, elastic net, lasso). This random generalized linear model (RGLM) predictor provides variable importance measures that can be used to define a “thinned” ensemble predictor (involving few features) that retains excellent predictive accuracy.

**Conclusion:**

RGLM is a state of the art predictor that shares the advantages of a random forest (excellent predictive accuracy, feature importance measures, out-of-bag estimates of accuracy) with those of a forward selected generalized linear model (interpretability). These methods are implemented in the freely available R software package *randomGLM*.

## Background

Prediction methods (also known as classifiers, supervised machine learning methods, regression models, prognosticators, diagnostics) are widely used in biomedical research. For example, reliable prediction methods are essential for accurate disease classification, diagnosis and prognosis. Since prediction methods based on multiple features (also known as covariates or independent variables) can greatly outperform predictors based on a single feature [1], it is important to develop methods that can optimally combine features to obtain high accuracy. Introductory text books describe well known prediction methods such as linear discriminant analysis (LDA), K-nearest neighbor (KNN) predictors, support vector machines (SVM) [2], and tree predictors [3]. Many publications have evaluated popular prediction methods in the context of gene expression data [4-9].

Ensemble predictors are particularly attractive since they are known to lead to highly accurate predictions. An ensemble predictor generates and integrates multiple versions of a single predictor (often referred to as base learner), and arrives at a final prediction by aggregating the predictions of multiple base learners, e.g. via plurality voting across the ensemble. One particular approach for constructing an ensemble predictor is bootstrap aggregation (bagging) [10]. Here multiple versions of the original data are generated through bootstrapping, where observations from the training set are randomly sampled with replacement. An individual predictor (e.g. a tree predictor) is fitted on each bootstrapped data set. Thus, 100 bootstrapped data sets (100 bags) will lead to an ensemble of 100 tree predictors. In case of a class outcome (e.g. disease status), the individual predictors “vote” for each class and the final prediction is obtained by majority voting.

Breiman (1996) showed that bagging weak predictors (e.g. tree predictors or forward selected linear models) often yields substantial gains in predictive accuracy [10]. But it seems that ensemble predictors are only very rarely used for predicting clinical outcomes. This fact points to a major weakness of ensemble predictors: they typically lead to ”black box” predictions that are hard to interpret in terms of the underlying features. Clinicians and epidemiologists prefer forward selected regression models since the resulting predictors are highly interpretable: a linear combination of relatively few features can be used to predict the outcome or the probability of an outcome. But the sparsity afforded by forward feature selection comes at an unacceptably high cost: forward variable selection (and other variable selection methods) often greatly overfit the data which results in unstable and inaccurate predictors [11,12]. Ideally, one would want to combine the advantages of ensemble predictors with those of forward selected regression models. As discussed below, multiple articles describe ensemble predictors based on linear models including the seminal work by Breiman [10] who evaluated a bagged forward selected linear regression model. However, the idea of bagging forward selected linear models (or other GLMs) appears to have been set aside as new ensemble predictors, such as the random forest, became popular. A random forest (RF) predictor not only bags tree predictors but also introduces an element of randomness by considering only a randomly selected subset of features at each node split [13]. The number of randomly selected features, *mtry*, is the only parameter of the random forest predictor. The random forest predictor has deservedly received a lot of attention for the following reasons: First, the bootstrap aggregation step allows one to use out-of-bag (OOB) samples to estimate the predictive accuracy. The resulting OOB estimate of the accuracy often obviates the need for cross-validation and other resampling techniques. Second, the RF predictor provides several measures of feature (variable) importance. Several articles explore the use of these importance measures to select genes [5,13,14]. Third, it can be used to define a dissimilarity measure that can be used in clustering applications [13,15]. Fourth, and most importantly, the RF predictor has superior predictive accuracy. It performs as well as alternatives in cancer gene expression data sets [5] but it really stands out when applied to the UCI machine learning benchmark data sets where it is as good as (if not better than) many existing methods [13]. While we confirm the truly outstanding predictive performance of the RF, the proposed RGLM method turns out to be even more accurate than the RF (e.g. across the disease gene expression data sets). Breiman and others have pointed out that the black box predictions of the RF predictor can be difficult to interpret. For this reason, we wanted to give bagged forward selected generalized linear regression models another careful look. After exploring different approaches for injecting elements of randomness into the individual GLM predictors, we arrived at a new ensemble predictor, referred to as random GLM predictor, with an astonishing predictive performance. An attractive aspect of the proposed RGLM predictor is that it combines the advantages of the RF with that of a forward selected GLM. As the name *generalized* linear model indicates, it can be used for a general outcome such as a binary outcome, a multi-class outcome, a count outcome, and a quantitative outcome. We show that several incremental (but important) changes to the original bagged GLM predictor by Breiman add to up to a qualitatively new predictor (referred to as random GLM predictor) that performs at least as well as the RF predictor on the UCI benchmark data sets. While the UCI data are the benchmark data for evaluating predictors, only a dozen such data sets are available for binary outcome prediction. To provide a more comprehensive empirical comparison of the different prediction methods, we also consider over 700 comparisons involving gene expression data. In these genomic studies, the RGLM method turns out to be slightly more accurate than the considered alternatives. While the improvements in accuracy afforded by the RGLM are relatively small they are statistically significant.

This article is organized as follows. First, we present a motivating example that illustrates the high prediction accuracy of the RGLM. Second, we compare the RGLM with other state of the art predictors when it comes to binary outcome prediction. Toward this end, we use the UCI machine learning benchmark data, over 700 empirical gene expression comparisons, and extensive simulations. Third, we compare the RGLM with other predictors for quantitative (continuous) outcome prediction. Fourth, we describe several variable importance measures and show how they can be used to define a thinned version of the RGLM that only uses few important features. Even for data sets comprised of thousands of gene features, the thinned RGLM often involves fewer than 20 features and is thus more interpretable than most ensemble predictors.

## Methods

### Construction of the RGLM predictor

RGLM is an ensemble predictor based on bootstrap aggregation (bagging) of generalized linear models whose features (covariates) are selected using forward regression according to AIC criterion. GLMs comprise a large class of regression models, e.g. linear regression for a normally distributed outcome, logistic regression for binary outcome, multi-nomial regression for multi-class outcome and Poisson regression for count outcome [16]. Thus, RGLM can be used to predict binary-, continuous-, count-, and other outcomes for which generalized linear models can be defined. The “randomness” in RGLM stems results from two sources. First, a non-parametric bootstrap procedure is used which randomly selects samples with replacement from the original data set. Second, a random subset of features (specified by input parameter *nFeaturesInBag*) is selected for each bootstrap sample. This amounts to a random sub-space method [17] applied to each bootstrap sample separately.

**Figure 1:**
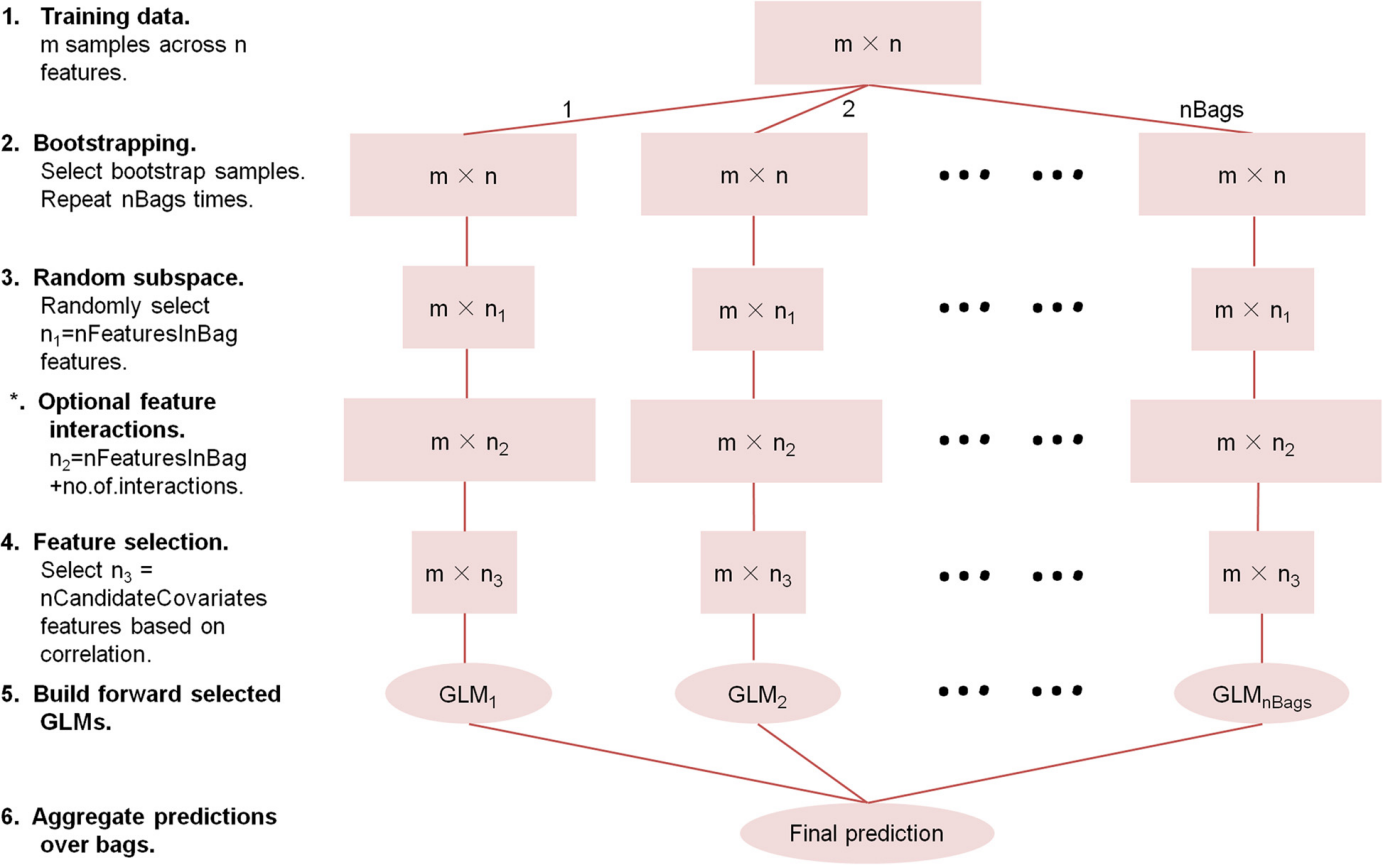
**Overview of the RGLM construction.** The figure outlines the steps used in the construction of the RGLM. The pink rectangles represent data matrices at each step. Width of a rectangle reflects the number of remaining features.

The steps of the RGLM construction are presented in Figure 1. First, starting from the original data set another equal-sized data set is generated using the non-parametric bootstrap method, i.e. samples are selected with replacement from the original data set. The parameter *nBags* (default value 100) determines how many of such bootstrap data sets (referred to as bags) are being generated. Second, a random set of features (determined by the parameter *nFeaturesInBag*) is randomly chosen for each bag. Thus, the GLM predictor for bag 1 will typically involve a different set of features than that for bag 2. Third, the *nFeaturesInBag* of randomly selected features per bag are rank-ordered according to their individual association with the outcome variable *y* in each bag. For a quantitative outcome *y*, one can simply use the absolute value of the correlation coefficient between the outcome and each feature to rank the features. More generally, one can fit a univariate GLM model to each feature to arrive at an association measure (e.g. a Wald test statistic or a likelihood ratio test). Only the top ranking features (i.e. features with the most significant univariate significance levels) will become candidate covariates for forward selection in a multivariate regression model. The top number of candidate features is determined by the input parameter *nCandidateCovariates* (default value 50). Fourth, forward variable (feature) selection is applied to the *nCandidateCovariates* of each bag to arrive at a multivariable generalized linear model per bag. The forward selection procedure used by RGLM is based on the *stepAIC* R function in the *MASS* R library where method is set to “forward”. Fifth, the predictions of each forward selected multivariate model (one per bag) are aggregated across bags to arrive at a final ensemble prediction. The aggregation method depends on the type of outcome. For a continuous outcome, predicted values are simply averaged across bags. For a binary outcome, the adjusted Majority Vote (aMV) strategy [18] is used which averages predicted probabilities across bags. Given the estimated class probabilities one can get a binary prediction by choosing an appropriate threshold (default=0.5).

Importantly, RGLM also has a parameter *maxInteractionOrder* (default value 1) for creating interactions up to a given order among features in the model construction. For example, RGLM.inter2 results from setting *maxInteractionOrder*=2, i.e. considering pairwise (also known as 2-way) interaction terms. As example, consider the case when only pairwise interaction terms are used. For each bag a random set of features is selected (similar to the random subspace method, RSM) from the original covariates, i.e. covariates without interaction terms. Next, all pairwise interactions among the *nFeaturesInBag* randomly selected features are generated. Next, the usual RGLM candidate feature selection steps will be applied to the combined set of pairwise interaction terms and the *nFeaturesInBag* randomly selected features per bag resulting in *nCandidateCovariates* top ranking features per bag, which are subsequently subjected to forward feature selection.

These methods are implemented in our R software package *randomGLM* which allows the user to input a training set and optionally a test set. It automatically outputs out-of-bag estimates of the accuracy and variable importance measures.

### Parameter choices for the RGLM predictor

As discussed below, we find that it is usually sufficient to consider only *nBags*=100 bootstrap data sets. The default value for *nFeaturesInBag* depends on the total number of features. It is easier to explain it in terms of the proportion of features randomly selected per bag, *nFeaturesInBag/N*, where *N* is the total number of features of the training set. Apart from *N* it is also valuable to consider the **effective** number of features which equals the number of features *N* plus the number of interaction terms, e.g. *N*^∗^=*N*+*N*(*N*−1)/2 in case of pairwise interactions. Using this notation, the default value of nFeaturesInBag can be arrived at by solving equations presented in Table 1. These equations were found by empirically evaluating various choices of *nFeaturesInBag* values (e.g. $\sqrt{N},N/5,N/3,N/2,2N/3,N$). In particular, we found that in case of *N*^∗^<=10, then using all features (i.e setting *nFeaturesInBag*/*N*=1) is often a good choice, whereas if *N*^∗^>300 then setting *nFeaturesInBag*/*N*=0.2 works well. The default value *nFeaturesInBag*/*N*=1.0276−0.00276*N*^∗^ in the intermediate case (10<*N*^∗^<=300) results from fitting an interpolation line through the two points (10,1) and (300, 0.2). We find that RGLM is quite robust with respect to the parameter *nFeaturesInBag*. To limit the number of covariates considered in forward selection (which is computationally intensive), the default value of *nCandidateCovariates* is set to 50. Overall, the default values perform well in our simulations, empirical gene expression and machine learning benchmark studies. But we recommend to use the OOB estimate of predictive accuracy to inform the choice of the parameter values.

**Table 1:** Default setting of nFeaturesInBag

	**N**	**nFeaturesInBag/N**	**N**^**∗**^	**nFeaturesInBag/N**
**No interaction**	1−10	1	1−10	1
	11−300	1.0276−0.00276*N*	11−300	1.0276−0.00276*N*^∗^
	>300	0.2	>300	0.2
**2-way interaction**	1−4	1	1−10	1
	5−24	1.0276−0.00276*N*(*N*+1)/2	11−300	1.0276−0.00276*N*^∗^
	>24	0.2	>300	0.2
**3-way interaction**	1−3	1	1−10	1
	4−12	1.0276−0.00276(*N*^3^+5*N*)/6	11−300	1.0276−0.00276*N*^∗^
	>12	0.2	>300	0.2

This table shows the default values of *nFeaturesInBag* in terms of *nFeaturesInBag/N* for RGLM, RGLM.inter2 and RGLM.inter3. *N* is the total number of features of the training data. *N*^∗^ is the effective number of features which equals the number of features *N* plus the number of interaction terms. Formulas are shown in terms of both *N* and the corresponding *N*^∗^. 1.0276 and 0.00276 are obtained by interpolating a straight line between (10,1) and (300, 0.2).

### Relationship with related prediction methods

As discussed below, RGLM can be interpreted as a variant of a bagged predictor [10]. In particular, it is similar to the bagged forward linear regression model [10] but differs in the following aspects: 

1. RGLM allows for interaction terms between features which greatly improve the performance on some data sets (in particular the UCI benchmark data sets). We refer to RGLM involving two-way or three way interactions as RGLM.inter2 and RGLM.inter3, respectively.

2. RGLM has a parameter *nFeaturesInBag* that allows one to restrict the number of features used in each bootstrap sample. This parameter is conceptually related to the *mtry* parameter of the Random Forest predictor. In essence, this parameter allows one to use a random subspace method (RSM, [17]) in each bootstrap sample.

3. RGLM has a parameter *nCandidateCovariates* that allows one to restrict the number of features in forward regression, which not only has computational advantages but also introduces additional instability into the individual predictors, which is a desirable characteristic of an ensemble predictor.

4. RGLM optimizes the AIC criterion during forward selection.

5. RGLM has a “thinning threshold” parameter which allows one to reduce the number of features involved in prediction while maintaining good prediction accuracy. Since a thinned RGLM involves far fewer features, it facilitates the understanding how the ensemble arrives at its predictions.

RGLM is not only related to bagging but also to the random subspace method (RSM) proposed by [17]. In the RSM, the training set is also repeatedly modified as in bagging but this modification is performed in the feature space (rather than the sample space). In the RSM, a subset of features is randomly selected which amounts to restricting attention to a subspace of the original feature space. As one of its construction steps, RGLM uses a RSM on each bootstrap sample. Future research could explore whether random partitions as opposed to random subspaces would be useful for constructing an RGLM. Random partitions of the feature space are similar to random subspaces but they divide the feature space into mutually exclusive subspaces [19,20]. Random partition based predictors have been shown to perform well in high-dimensional data ([19]). Both RSM and random partitions have more general applicability than RGLM since these methods can be used for any base learner. There is a vast literature on ensemble induction methods but a property worth highlighting is that RGLM uses forward variable selection of GLMs. Recall that RGLM goes through the following steps: 1) bootstrap sampling, 2) RSM (and optionally creating interaction terms), 3) forward variable selection of a GLM, 4) aggregation of votes. Empirical studies involving different base learners (other than GLMs) have shown that combining bootstrap sampling with RSM (steps 1 and 2) leads to ensemble predictors with comparable performance to that of the random forest predictor [21].

Another prediction method, random multinomial logit model (RMNL), also shares a similar idea with RGLM. It was recently proposed for multi-class outcome prediction [18]. RMNL bags multinomial logit models with random feature selection in each bag. It can be seen as a special case of RGLM, except that no forward model selection is carried out.

### Software implementation

The RGLM method is implemented in the freely available R package *randomGLM*. The R function *randomGLM* allows the user to output training set predictions, out-of-bag predictions, test set predictions, coefficient values, and variable importance measures. The *predict* function can be used arrive at test set predictions. Tutorials can be found at the following webpage: http://labs.genetics.ucla.edu/horvath/RGLM.

### Short description of alternative prediction methods

#### 

**Forward selected generalized linear model predictor (forwardGLM)** We denote by *forwardGLM* the (single) generalized linear model predictor whose covariates were selected using forward feature selection (according to the AIC criterion). Thus, forwardGLM does not involve bagging, random feature selection, and is not an ensemble predictor.

#### 

**Random forest (RF)** RF is an ensemble predictor that consists of a collection of decision trees which vote for the class of observations [13]. The RF is known for its outstanding predictive accuracy. We used the *randomForest* R package in our studies. We considered two choices for the RF parameter *mtry*: i) the default RF predictor where *mtry* equals the square root of the number of features and ii) *RFbigmtry* where *mtry* equals the total number of features. We always generated at least 500 trees per forest but used 1000 trees when calculating variable importance measures.

#### 

**Recursive partitioning and regression trees (Rpart)** Classification and regression trees were generated using the default settings *rpart* R package. Tree methods are described in [3].

#### 

**Linear discriminant analysis (LDA)** LDA aims to find a linear combination of features (referred to as discriminant variables) to predict a binary outcome (reviewed in [22,23]). We used the *lda* R function in the *MASS* R package with parameter choice *method*=*moment*.

#### 

**Diagonal linear discriminant analysis (DLDA)** DLDA is similar to LDA but it ignores the correlation patterns between features. While this is often an unrealistic assumption, DLDA (also known as gene voting) has been found to work well in in gene expression applications [4]. Here we used the default parameters from the *supclust* R package [24].

#### 

**K nearest neighbor (KNN)** We used the *knn* R function in the *class* R package [22,23], which chose the parameter *k* of nearest neighbors using 3-fold cross validation (CV).

#### 

**Support vector machines (SVM)** We used the default parameters from the *e1071* R package to fit SVMs [2]. Additional details can be found in [25].

#### 

**Shrunken centroids (SC)** The SC predictor is known to work well in the context of gene expression data [26]. Here we used the implementation in the *pamr* R package [26] which chose the optimal level of shrinkage using cross validation.

#### 

**Penalized regression models** Various convex penalties can be applied to generalized linear models. We considered ridge regression [27] corresponding to an *ℓ*_2_ penalty, the lasso corresponding to an *ℓ*_1_ penalty [28], and elastic net corresponding to a linear combination of *ℓ*_1_ and *ℓ*_2_ penalties [29]. We used the *glmnet* R function from the *glmnet* R package [30,31] with alpha parameter values of 0, 1, and 0.5 respectively. *glmnet* also involves another parameter (lambda) which was chosen as the median of the lambda sequence output resulting from *glmnet*. For UCI benchmark data sets, pairwise interaction between features were considered.

### 20 disease-related gene expression data sets

We use 20 disease related gene expression data sets involving cancer and other human diseases (described in Table 2). The first 10 data sets involving various cancers were previously used by [5]. These data can be downloaded from the author’s webpage at http://ligarto.org/rdiaz/Papers/rfVS/randomForestVarSel.html. The BrainTumor2 and DLBCL data sets were downloaded from http://www.gems-system.org/. The remaining 8 data sets (lung1 – MSdiagnosis2) were downloaded from either the Gene Expression Omnibus (GEO) database or the ArrayExpress data base in raw form and subsequently preprocessed using MAS5 normalization and quantile normalization. Only the top 10000 probes (features) with highest mean expression were considered for outcome prediction. We briefly point out that Diaz *et al* (2006) report prediction error rates estimated using a bootstrap method. In contrast, we report 3-fold cross validation estimates (averaged over 100 random partitions of the data into 3 folds), which may explain minor numerical differences between our study and that of Diaz *et al* (2006).

**Table 2:** Description of the 20 disease expression data sets

**Data set**	**Samples**	**Features**	**Reference**	**Data set ID**	**Binary outcome**
**adenocarcinoma**	76	9868	[32]	NA	most prevalent class vs others
**brain**	42	5597	[33]	NA	most prevalent class vs others
**breast2**	77	4869	[34]	NA	most prevalent class vs others
**breast3**	95	4869	[34]	NA	most prevalent class vs others
**colon**	62	2000	[35]	NA	most prevalent class vs others
**leukemia**	38	3051	[36]	NA	most prevalent class vs others
**lymphoma**	62	4026	[37]	NA	most prevalent class vs others
**NCI60**	61	5244	[38]	NA	most prevalent class vs others
**prostate**	102	6033	[39]	NA	most prevalent class vs others
**srbct**	63	2308	[40]	NA	most prevalent class vs others
**BrainTumor2**	50	10367	[41]	NA	Anaplastic oligodendrogliomas vs Glioblastomas
**DLBCL**	77	5469	[42]	NA	follicular lymphoma vs diffuse large B-cell lymphoma
**lung1**	58	10000	[43]	GSE10245	Adenocarcinoma vs Squamous cell carcinoma
**lung2**	46	10000	[44]	GSE18842	Adenocarcinoma vs Squamous cell carcinoma
**lung3**	71	10000	[45]	GSE2109	Adenocarcinoma vs Squamous cell carcinoma
**psoriasis1**	180	10000	[46,47]	GSE13355	lesional vs healthy skin
**psoriasis2**	82	10000	[48]	GSE14905	lesional vs healthy skin
**MSstage**	26	10000	[49]	E-MTAB-69	relapsing vs remitting RRMS
**MSdiagnosis1**	27	10000	[50]	GSE21942	RRMS vs healthy control
**MSdiagnosis2**	44	10000	[49]	E-MTAB-69	RRMS vs healthy control

Sample size, number of features, original reference, data set IDs and outcomes for the 20 disease related gene expression data sets.

### Empirical gene expression data sets

For all data sets below, we considered 100 randomly selected gene traits, i.e. 100 randomly selected probes. They were directly used as continuous outcomes or dichotomized according to the median value (top half =1, bottom half =0) to generate binary outcomes. For all data sets except “Brain cancer”, $\frac{2}{3}$ of the observations (arrays) were randomly chosen as the training set, while the remaining samples were chosen as test set. We focused on the 5000 genes (probes) with the highest mean expression levels in each data set.

### 

**Brain cancer data sets** These two related data sets contain 55 and 65 microarray samples of glioblastoma (brain cancer) patients, respectively. Gene expression profiles were measured using Affymetrix U133 microarrays. A detailed description can be found in [51]. The first data set (comprised of 55 samples) was used as a training set while and the second data set (comprised of 65 samples) was used as a test set.

### 

**SAFHS blood lymphocyte data set** This data set [52] was derived from blood lymphocytes of randomly ascertained participants enrolled in the San Antonio Family Heart Study. Gene expression profiles were measured with the Illumina Sentrix Human Whole Genome (WG-6) Series I BeadChips. After removing potential outliers (based on low interarray correlations), 1084 samples remained in the data set.

### 

**WB whole blood gene expression data set** This is the whole blood gene expression data from healthy controls. Peripheral blood samples from healthy individuals were analyzed using Illumina Human HT-12 microarrays. After pre-processing, 380 samples remained in the data set.

### 

**Mouse tissue gene expression data sets** The 4 tissue specific gene expression data sets were generated by the lab of Jake Lusis at UCLA. These data sets measure gene expression levels (Agilent array platform) from adipose (239 samples), brain (221 samples), liver (272 samples) and muscle (252 samples) tissue of mice from the B ×H *F*_2_ mouse intercross described in [53,54]. In addition to gene traits, we also predicted 21 quantitative mouse clinical traits including mouse weight, length, abdominal fat, other fat, total fat, adiposity index (total fat ∗100/weight), plasma triglycerides, total plasma cholesterol, high-density lipoprotein fraction of cholesterol, plasma unesterified cholesterol, plasma free fatty acids, plasma glucose, plasma low-density lipoprotein and very low-density lipoprotein cholesterol, plasma MCP-1 protein levels, plasma insulin, plasma glucose-insulin ratio, plasma leptin, plasma adiponectin, aortic lesion size (measured by histological examination using a semi-quantitative scoring methods), aneurysms (semi-quantitative scoring method), and aortic calcification in the lesion area.

### Machine learning benchmark data sets

The 12 machine learning benchmark data sets used in this article are listed in Table 3. Note that only eight of the 12 data sets have a binary outcomes. The multi-class outcomes of the 4 remaining data sets were turned into binary outcomes by considering the most prevalent class versus all other classes combined. Missing data were imputed using nearest neighbor averaging. For each data set and prediction method, we report the average 3-fold CV estimate of prediction accuracy over 100 random partitions of the data into 3 folds.

**Table 3:** Description of the UCI benchmark data

**Data set**	**Samples**	**Features**
**BreastCancer**	699	9
**HouseVotes84**	435	16
**Ionosphere**	351	34
**diabetes**	768	8
**Sonar**	208	60
**ringnorm**	300	20
**threenorm**	300	20
**twonorm**	300	20
**Glass**	214	9
**Satellite**	6435	36
**Vehicle**	846	18
**Vowel**	990	10

Sample size and number of features for the 12 UCI machine learning benchmark data sets.

### Simulated gene expression data sets

We simulated an outcome variable *y* and gene expression data that contained 5 modules (clusters). Only 2 of the modules were comprised of genes that correlated with the outcome *y*. 45% of the genes were background genes, i.e. these genes were outside of any module. The simulation scheme is detailed in Additional file 1 and implemented in the R function *simulateDatExpr5Modules* from the *WGCNA* R package [55]. This R function was used to simulate pairs of training and test data sets. The simulation study was used to evaluate prediction methods for continuous outcomes and for binary outcomes. For binary outcome prediction, the continuous outcome *y* was thresholded according to its median value.

We considered 180 different simulation scenarios involving varying sizes of the training data (50, 100, 200, 500, 1000 or 2000 samples) and varying numbers of genes (60, 100, 500, 1000, 5000 or 10000 genes) that served as features. Test sets contained the same number of genes as in the corresponding training set and 1000 samples. For each simulation scenario, we simulate 5 replicates resulting from different choices of the random seed.

## Results

### Motivating example: disease-related gene expression data sets

We compare the prediction accuracy of RGLM with that of other widely used methods on 20 gene expression data sets involving human disease related outcomes. Many of the 20 data sets (Table 2) are well known cancer data sets, which have been used in other comparative studies [4,5,56,57]. A brief description of the data sets can be found in Methods.

To arrive at an unbiased estimate of prediction accuracy, we used 3-fold cross validation (averaged over 100 random partitions of the data into 3 folds). Note that the accuracy equals 1 minus the median misclassification error rate. Table 4 reports the prediction accuracy of different methods including RGLM, random forest (RF, with default value for its *mtry* parameter), random forest (RFbigmtry, with *mtry* equal to the total number of features), tree predictor (also known as recursive partitioning, Rpart), linear discriminant analysis (LDA), diagonal linear discriminant analysis (DLDA), k nearest neighbor (KNN), support vector machine (SVM) and shrunken centroid (SC). A short description of these prediction methods is provided in Methods.

**Table 4:** Prediction accuracy in the 20 disease gene expression data sets

**Data set**	**RGLM**	**RF**	**RFbigmtry**	**Rpart**	**LDA**	**DLDA**	**KNN**	**SVM**	**SC**
**adenocarcinoma**	0.842	0.842	0.842	0.737	0.842	0.744	0.842	0.842	0.803
**brain**	0.881	0.810	0.833	0.762	0.810	0.929	0.881	0.786	0.929
**breast2**	0.623	0.610	0.636	0.584	0.610	0.636	0.584	0.558	0.636
**breast3**	0.705	0.695	0.716	0.611	0.695	0.705	0.669	0.674	0.700
**colon**	0.855	0.823	0.823	0.726	0.855	0.839	0.774	0.774	0.871
**leukemia**	0.921	0.895	0.921	0.816	0.868	0.974	0.974	0.763	0.974
**lymphoma**	0.968	1.000	1.000	0.903	0.960	0.984	0.984	1.000	0.984
**NCI60**	0.902	0.869	0.869	0.738	0.885	0.902	0.852	0.869	0.918
**prostate**	0.931	0.892	0.902	0.853	0.873	0.627	0.804	0.853	0.912
**srbct**	1.000	0.944	0.984	0.921	0.857	0.905	0.952	0.873	1.000
**BrainTumor2**	0.760	0.750	0.740	0.620	0.760	0.700	0.700	0.660	0.720
**DLBCL**	0.909	0.851	0.883	0.831	0.922	0.779	0.870	0.792	0.857
**lung1**	0.931	0.931	0.931	0.828	0.914	0.931	0.931	0.897	0.914
**lung2**	0.935	0.935	0.935	0.826	0.957	0.978	0.935	0.848	0.978
**lung3**	0.901	0.901	0.887	0.803	0.873	0.859	0.831	0.859	0.887
**psoriasis1**	0.989	0.994	0.989	0.978	0.994	0.989	0.989	0.983	0.989
**psoriasis2**	0.963	0.988	0.976	0.963	0.976	0.963	0.963	0.963	0.963
**MSstage1**	0.846	0.846	0.846	0.423	0.769	0.769	0.808	0.769	0.769
**MSdiagnosis1**	0.963	0.926	0.926	0.556	0.889	0.889	0.963	0.926	0.926
**MSdiagnosis2**	0.591	0.614	0.614	0.568	0.545	0.568	0.568	0.568	0.523
**MeanAccuracy**	0.871	0.856	0.863	0.752	0.843	0.833	0.844	0.813	0.863
**Rank**	1	4	2.5	9	6	7	5	8	2.5
**Pvalue**	NA	0.029	0.079	0.00014	0.0075	0.05	0.014	0.00042	0.37

For each data set, the prediction accuracy was estimated using 3−*fold* cross validation across 100 random partitions of the data into 3 folds. Mean accuracies across the 20 data sets and the resulting ranks are summarized at the bottom. Two sided paired Wilcoxon test p-values can be used to determine whether the accuracy of RGLM is significantly different from that of other predictors. Note that the RGLM yields the highest mean accuracy.

As seen from Table 4, RGLM achieves the highest mean accuracy in these disease data sets, followed by RFbigmtry and SC. Note that the standard random forest predictor (with default parameter choice) performs worse than RGLM. The accuracy difference between RGLM and alternative methods is statistically significant (Wilcoxon signed rank test <0.05) for all predictors except for RFbigmtry, DLDA and SC. Since RFbigmtry is an ensemble predictor that relies on thousands of features it would be difficult to interpret its predictions in terms of the underlying genes.

Our evaluations focused on the accuracy (and misclassification error). However, a host of other accuracy measures could be considered. Additional file 2 presents the results for sensitivity and specificity. The top 3 methods with highest sensitivity are: RF (median sensitivity =0.969), SVM (0.969) and RGLM (0.960). The top 3 methods with highest specificity are: SC (0.900), RGLM (0.857) and KNN (0.848).

A strength of this empirical comparison is that it involves clinically or biologically interesting data sets but a severe limitation is that it only involves 20 comparisons. Therefore, we now turn to more comprehensive empirical comparisons.

### Binary outcome prediction

#### Empirical study involving dichotomized gene traits

**Figure 2:**
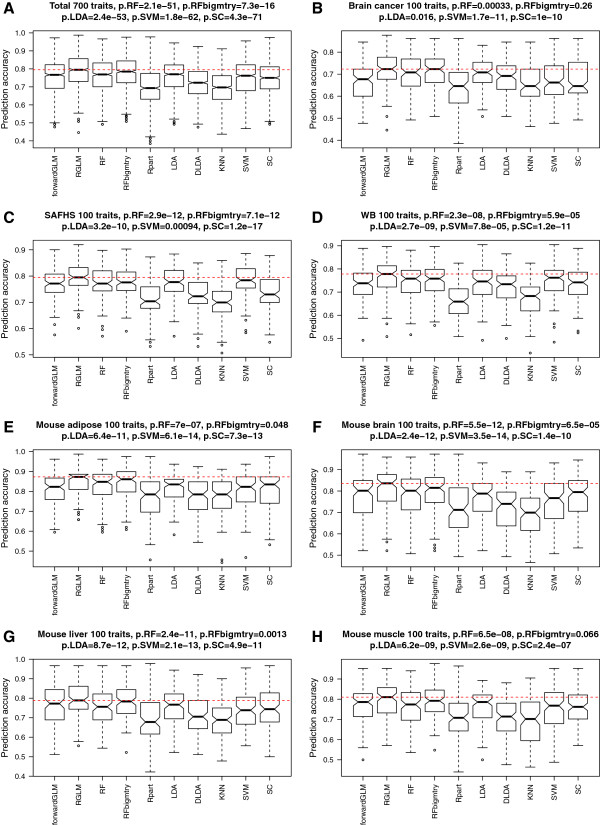
**Binary outcome prediction in empirical gene expression data sets.** The boxplots show the test set prediction accuracies across 700 comparisons. The horizontal line inside each box represents the median accuracy. The horizontal dashed red line indicates the median accuracy of the RGLM predictor. P-values result from using the two-sided Wilcoxon signed rank test for evaluating whether the median accuracy of RGLM is the same as that of the mentioned method. For example, p.RF results from testing whether the median accuracy of RGLM is the same as that of the RF. **(A)** summarizes the test set performance for predicting 100 dichotomized gene traits from each of the 7 expression data sets. **(B-H)** show the results for individual data sets. 100 randomly chosen, dichotomized gene traits were used. Note the superior accuracy of the RGLM predictor across the different data sets.

Many previous empirical comparisons of gene expression data considered fewer than 20 data sets. To arrive at 700 comparisons, we use the following approach: We started out with 7 human and mouse gene expression data sets. For each data set, we randomly chose 100 genes as gene traits (outcomes) resulting in 7×100 possible outcomes. We removed the gene corresponding to the gene trait from the feature set. Next, each gene trait was dichotomized by its median value to arrive at a binary outcome *y*. The goal of each prediction analysis was to predict the dichotomized gene trait *y* based on the other genes. At first sight, this artificial outcome is clinically uninteresting but it is worth emphasizing that clinicians often deal with dichotomized measures of gene products, e.g. high serum creatinine levels may indicate kidney damage, high PSA levels may indicate prostate cancer, and high HDL levels may indicate hypercholesterolemia. To arrive at unbiased estimates of prediction accuracy, we split each data set into a training and test set. Figure 2 (A) shows boxplots of the accuracies across the 700 comparisons. Similar performance patterns are observed for the individual data sets (Figure 2 (B-H)). The figure also reports pairwise comparisons of the RGLM method versus alternative methods. Specifically, it reports the two-sided Wilcoxon signed rank test p-values for testing whether the accuracy of the RGLM predictor is higher than that of the considered alternative method. Strikingly, RGLM is more accurate than the other methods overall. While the increase in accuracy is often minor, it is statistically significant as can be seen by comparing RGLM to RF (median difference =0.02,*p*=2.1×10^−51^), RFbigmtry (median difference =0.01,*p*=7.3×10^−16^), LDA (median difference =0.06,*p*=2.4×10^−53^), SVM (median difference =0.03,*p*=1.8×10^−62^) and SC (median difference =0.04,*p*=4.3×10^−71^). Other predictors perform even worse, and the corresponding p-values are not shown.

The fact that RFbigmtry is more accurate in this situation than the default version of RF probably indicates that relatively few genes are informative for predicting a dichotomized gene trait. Also note that RGLM is much more accurate than the unbagged forward selected GLM which reflects that forward selection greatly overfits the training data. In conclusion, these comprehensive gene expression studies show that RGLM has outstanding prediction accuracy.

#### Machine learning benchmark data analysis

Here we evaluate the performance of RGLM on the UCI machine learning benchmark data sets which are often used for evaluating prediction methods [10,13,58-61]. We consider 12 benchmark data sets from the *mlbench* R package: 9 UCI data sets and 3 synthetic data sets (Table 3). We choose these data sets for two reasons. First, these 12 data sets were also used in the original evaluation of the random forest predictor [13]. Second, these data include all of the available data sets with binary outcomes in the *mlbench* R package. A detailed description of these data sets can be found in Methods. In his original publication on the random forest, Breiman found that the RF outperformed bagged predictors on the UCI benchmark data which may explain why bagged GLMs have not received much attention. We hypothesize that the relatively poor performance of a bagged logistic regression model on these data sets could be ameliorated by considering interaction terms between the features. Table 5 confirms our hypothesis. RGLM.inter2 (corresponding to pairwise interaction terms) has superior or tied accuracy compared to RGLM in 10 out of 12 benchmark data sets. In particular, pairwise interactions greatly improve the prediction accuracy in the ringnorm data set. Higher order interactions (RGLM.inter3) do not perform better than RGLM.inter2 but dramatically increase computational burden (data not shown).

**Table 5:** Prediction accuracy in the UCI machine learning benchmark data

**Data set**	**RGLM**	**RGLM.inter2**	**RF**	**RFbigmtry**	**Rpart**	**LDA**	**DLDA**	**KNN**	**SVM**	**SC**
**BreastCancer**	0.964	0.959	0.969	0.961	0.941	0.957	0.959	0.966	0.967	0.956
**HouseVotes84**	0.961	0.963	0.958	0.954	0.954	0.951	0.914	0.924	0.958	0.938
**Ionosphere**	0.883	0.946	0.932	0.917	0.875	0.863	0.809	0.849	0.940	0.829
**diabetes**	0.768	0.759	0.759	0.754	0.741	0.768	0.732	0.740	0.757	0.743
**Sonar**	0.769	0.837	0.817	0.788	0.707	0.726	0.697	0.812	0.822	0.726
**ringnorm**	0.577	0.973	0.940	0.910	0.770	0.567	0.570	0.590	0.977	0.535
**threenorm**	0.803	0.827	0.807	0.777	0.653	0.817	0.825	0.815	0.853	0.817
**twonorm**	0.937	0.953	0.947	0.920	0.733	0.957	0.960	0.947	0.953	0.960
**Glass**	0.636	0.743	0.827	0.799	0.729	0.659	0.531	0.808	0.748	0.645
**Satellite**	0.986	0.987	0.988	0.985	0.961	0.985	0.734	0.990	0.988	0.803
**Vehicle**	0.965	0.986	0.986	0.973	0.944	0.967	0.729	0.909	0.974	0.752
**Vowel**	0.936	0.986	0.983	0.976	0.950	0.938	0.853	0.999	0.991	0.909
**MeanAccuracy**	0.849	0.910	0.909	0.893	0.830	0.846	0.776	0.862	0.911	0.801
**Rank**	6	2	2	4	8	7	10	5	2	9
**Pvalue**	0.0093	NA	0.26	0.042	0.00049	0.0093	0.0067	0.11	0.96	0.0015

For each data set, the prediction accuracy was estimated using 3−*fold* cross validation across 100 random partitions of the data into 3 folds. RGLM.inter2 incorporates pairwise interaction between features into the RGLM predictor. Mean accuracies and the resulting ranks are summarized at the bottom. The Wilcoxon signed rank test was used to test whether accuracy differences between RGLM.inter2 and other predictors are significant. RGLM.inter2, RF, and SVM tie for first place (resulting in a rank of 2 for each method).

Overall, we find that RGLM.inter2 ties with SVM (*diff* =−0.001,*p*=0.96) and RF (*diff* =0.001,*p*=0.26) for the first place in the benchmark data. As can be seen from Additional file 3, RGLM.inter2 achieves the highest sensitivity and specificity, which also support its good performance in the benchmark data sets.

A potential limitation of these comparisons is that we considered pairwise interaction terms for the RGLM predictor but not for the other predictors. To address this issue, we also considered pairwise interactions among features for other predictors. Additional file 4 shows that no method surpasses RGLM.inter2 when pairwise interaction terms are considered. In particular, interaction terms between features do not improve the performance of the random forest predictor. A noteworthy disadvantage of RGLM.inter in case of many features is the computational burden that may result from adding interaction terms. In applications where interaction terms are needed for RGLM, faster alternatives (e.g. RF) remain an attractive choice.

#### Simulation study involving binary outcomes

**Figure 3:**
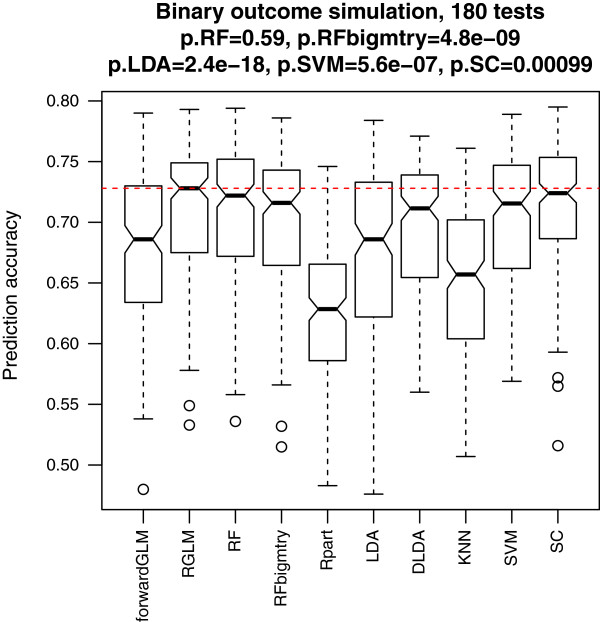
**Binary outcome prediction in simulation.** This boxplot shows the test set prediction accuracies across the 180 simulation scenarios.The red dashed line indicates the median accuracy of the RGLM. P-values result from using the two-sided Wilcoxon signed rank test for evaluating whether the median accuracy of RGLM is the same as that of the mentioned method.

As described in Methods, we simulated 180 gene expression data sets with binary outcomes. The number of features (genes) ranged from 60 to 10000. The sample sizes (number of observations) of the training data ranged from 50 to 2000. To robustly estimate the test set accuracy we chose a large size for the corresponding test set data, *n*=1000. Figure 3 shows the boxplots of the test set accuracies of different predictors. The accuracy of the forwardGLM is much lower than that of RGLM, demonstrating the benefit of creating an ensemble predictor. The Wilcoxon test p-value shows that RGLM is significantly better than all other methods except for the RF (no significant difference). In this simulation study, RGLM takes the first place when it comes to the median accuracy.

### Continuous outcome prediction

In the following, we show that RGLM also performs exceptionally well when dealing with continuous quantitative outcomes. We not only compare RGLM to a standard forward selected linear model predictor (forwardGLM) but also a random forest predictor (for a continuous outcome). We do not report the findings for the k-nearest neighbor predictor of a continuous outcome since it performed much worse than the above mentioned approaches in our gene expression applications (the accuracy of a KNN predictor was decreased by about 30 percent). We again split the data into training and test sets. We use the correlation between test set predictions and truly observed test set outcomes as measure of predictive accuracy. Note that this correlation coefficient can take on negative values (in case of a poorly performing prediction method).

#### Empirical study involving continuous gene traits

**Figure 4:**
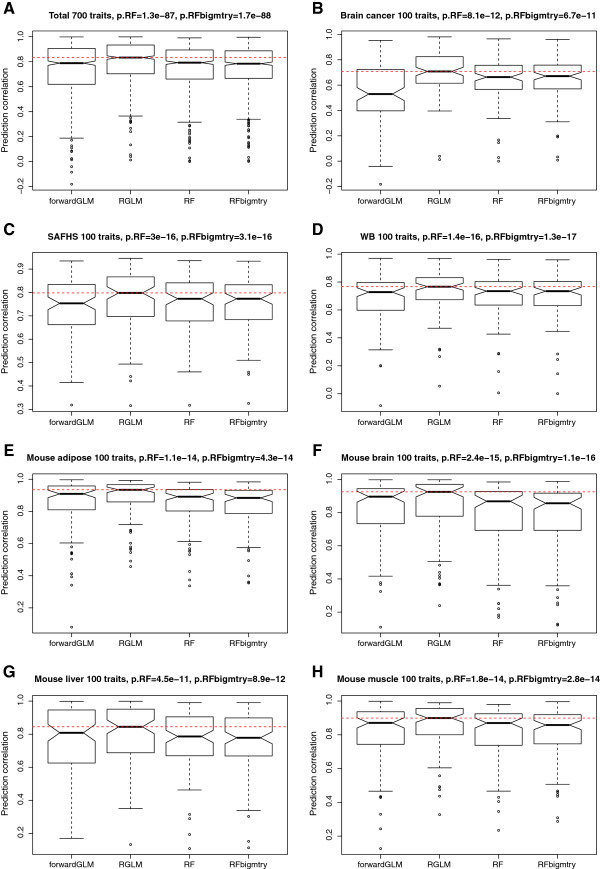
**Continuous outcome prediction in empirical gene expression data sets.** The boxplots show the test set prediction correlation in 700 applications. P-values result from using the two-sided Wilcoxon signed rank test for evaluating whether the median accuracy of RGLM is the same as that of the mentioned method. **(A)** summarizes the test set performance for predicting 100 continuous gene traits from each of the 7 expression data set. **(B-H)** show the results for individual data sets. RGLM is superior to other methods overall.

Here we used the same 700 gene expression comparisons as described above (100 randomly chosen gene traits from each of 7 gene expression data sets) but did not dichotomize the gene traits. Incidentally, prediction methods for gene traits are often used for imputing missing gene expression values. Our results presented in Figure 4 indicate that for the majority of genes high accuracies can be achieved. But for some gene traits, the accuracy measure, which is defined as a correlation coefficient, takes on negative values indicating that there is no signal in the data. Note that the forward selected linear predictor ties with the random forest irrespective of the choice of the *mtry* parameter and both methods perform significantly worse than the RGLM predictor.

#### Mouse tissue expression data involving continuous clinical outcomes

**Figure 5:**
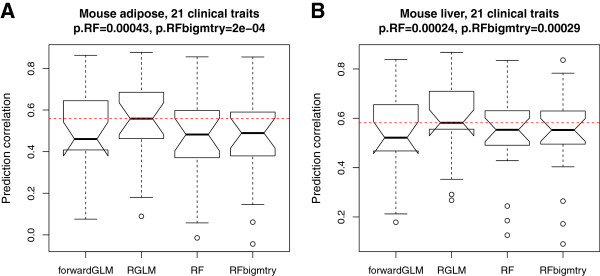
**Continuous clinical outcome prediction in mouse adipose and liver data sets.** The boxplots show the test set prediction correlation for predicting 21 clinical outcomes in **(A)** mouse adipose and **(B)** mouse liver. The red dashed line indicates the median correlation for RGLM. P-values result from using the two-sided Wilcoxon signed rank test for evaluating whether the median accuracy of RGLM is the same as that of the mentioned method.

Here we used the mouse liver and adipose tissue gene expression data sets to predict 21 clinical outcomes (detailed in Methods). Again, RGLM achieved significantly higher median prediction accuracy compared to the other predictors (Figure 5).

#### Simulation study involving continuous outcomes

**Figure 6:**
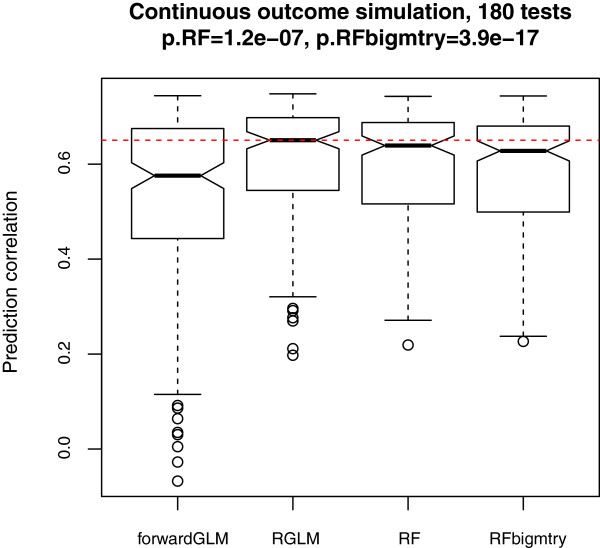
**Continuous outcome prediction in simulation studies.** This boxplot shows the test set prediction accuracy across the 180 simulation scenarios. The red dashed line indicates the median accuracy for the RGLM. Wilcoxon signed rank test p-values are presented.

180 gene expression data sets are simulated in the same way as described previously (for evaluating a binary outcome) but here the outcome *y* was not dichotomized. As shown in Figure 6, the forwardGLM accuracy trails both RGLM and RF, reflecting again the fact that forward regression overfits the data. In this simulation study, we find that RGLM yields significantly higher prediction accuracy than other predictors.

### Comparing RGLM with penalized regression models

**Figure 7:**
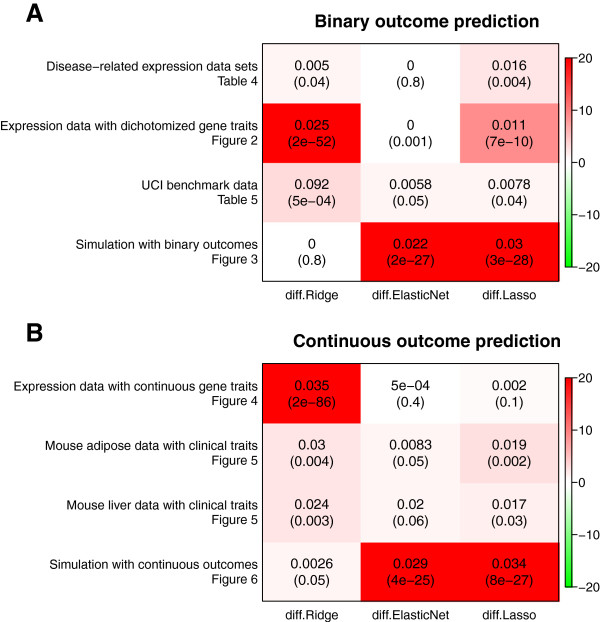
**Penalized regression models versus RGLM.** The heatmap reports the median difference in accuracy between RGLM and 3 types of penalized regression models in **(A)** binary outcome prediction and **(B)** continuous outcome prediction. Each cell entry reports the paired median difference in accuracy (upper number) and the corresponding Wilcoxon signed rank test p-value (lower number). The cell color indicates the significance of the finding, where red implies that RGLM outperforms penalized regression model and green implies the opposite. The color panel on the right side shows how colors correspond to −*log*10(p-values). *diff.Ridge* =*median(RGLM.accuracy* −*RidgeRegression.accuracy)*. *diff.ElasticNet* =*median(RGLM.accuracy* −*ElasticNet.accuracy)*. *diff.Lasso* =*median(RGLM.accuracy* −*Lasso.accuracy)*.

In our previous comparisons, we found that RGLM greatly outperforms forward selected GLM methods based on the AIC criterion. Many powerful alternatives to forward variable selection have been developed in the literature, in particular penalized regression models. Here, we compare RGLM to 3 major types of penalized regression models: ridge regression [27], elastic net [29], and the lasso [28]. The predictive accuracies of these penalized regression models were compared to those of the RGLM predictor using the same data sets described above for evaluating binary outcome and quantitative outcome prediction methods. Wilcoxon’s signed rank test was used to determine whether differences in predictive accuracy were significant. Figure 7 (A) shows that RGLM outperforms penalized regression models when applied to binary outcomes. For all comparisons, the paired median difference (median of RGLM accuracy minus penalized regression accuracy) is positive which indicates that RGLM is at least as good if not better than any of these 3 penalized regression models. In particular, RGLM is significantly better than ridge regression (*diff* =0.025,*p*=2×10^−52^) and the lasso (*diff* =0.011,*p*=7×10^−10^) on the 700 dichotomized gene expression trait data. Also, RGLM is significantly better than elastic net (*diff* =0.022,*p*=2×10^−27^) and lasso (*diff* =0.03,*p*=3×10^−28^) in simulations with binary outcomes. Figure 7 (B) shows that RGLM outperforms penalized regression models for continuous outcome prediction as well. Positive accuracy differences again imply that RGLM is at least as good as these penalized regression models. In particular, it significantly outperforms ridge regression (*diff* =0.035,*p*=2×10^−86^) in the 700 continuous gene expression traits data and outperforms elastic net (*diff* =0.029,*p*=4×10^−25^) and lasso (*diff* =0.034,*p*=8×10^−27^) in simulations with continuous outcomes.

As a caveat, we mention that cross validation methods were not used to inform the parameter choices of the penalized regression models since the RGLM predictor was also not allowed to fine tune its parameters. By only using default parameter choices we ensure a fair comparison. In a secondary analysis, however, we allowed penalized regression models to use cross validation for informing the choice of the parameters. While this slightly improved the performance of the penalized regression models (data not shown), it did not affect our main conclusion. RGLM outperforms penalized regression models in these comparisons.

### Feature selection

Here we briefly describe how RGLM naturally gives rise to variable (feature) importance measures. We compare the variable importance measures of RGLM with alternative approaches and show how variable importance measures can be used for defining a thinned RGLM predictor with few features.

#### Variable importance measure

There is a vast literature on using ensemble predictors and bagging for selecting features. For example, Meinshausen and Bühlmann describe “stability selection” based on variable selection employed in regression models [62]. The method involves repetitive sub-sampling, and variables that occur in a large fraction of the resulting selection set are chosen. Li et al. use a random k-nearest neighbor predictor (RKNN) to carry out feature selection [57]. The Entropy-based Recursive Feature Elimination (E-RFE) method of Furlanello et al. ranks features in high dimensional microarray data [63]. RGLM, like many ensemble predictors, gives rise to several measures of feature (variable) importance. For example, the number of times a feature is selected in the forward GLM across bags, *timesSelectedByForwardRegression*, is a natural measure of variable importance (similar to that used in stability selection [62]). Another variable importance measure is the number of times a feature is selected as candidate covariate for forward regression, *timesSelectedAsCandidates*. Note that both *timesSelectedByForwardRegression* and *timesSelectedAsCandidates* have to be ≤*nBags*. Finally, one can use the sum of absolute GLM coefficient values, *sumAbsCoefByForwardRegression*, as a variable importance measure. We prefer *timesSelectedByForwardRegression*, since it is more intuitive and points to the features that directly contribute to outcome prediction.

**Figure 8:**
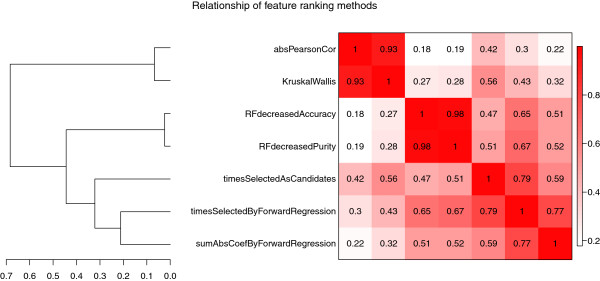
**Relationship between variable importance measures based on the Pearson correlation across 70 tests.** This figure shows the hierarchical cluster tree (dendrogram) of 7 variable importance measures. *absPearsonCor* is the absolute Pearson correlation between each gene and the dichotomous trait. *KruskalWallis* stands for the −*log*10 p-value of the Kruskal-Wallis group comparison test (which evaluates whether the gene is differentially expressed between the two groups defined by the binary trait). *RFdecreasedAccuracy* and *RFdecreasedPurity* are variable importance measures of the RF. *timesSelectedAsCandidates, timesSelectedByForwardRegression* and *sumAbsCoefByForwardRegression* are RGLM measures. These measures are evaluated in 10 tests from each of the 7 empirical expression data sets. In every test, different measures independently score genes for their relationship with a specific dichotomized gene trait. A Pearson correlation matrix was calculated by correlating the scores of different variable importance methods. Matrices across the 70 tests were averaged and the result was transformed to a dissimilarity measure that was subsequently used as input of hierarchical clustering.

To reveal relationships between different types of variable importance measures, we present a hierarchical cluster tree of RGLM measures, RF measures and standard marginal analysis based on correlations in Figure 8. As expected, the marginal association measures (standard Pearson correlation and the Kruskal-Wallis test which can both be used for a binary outcome) cluster together. The same holds for the random forest based importance measures (“mean decreased accuracy” and “mean decreased node purity”) and the 3 RGLM based importance measures.

Leo Breiman already pointed out that random forests could be used for feature selection in genomic applications. Díaz-Uriarte et al. proposed a related gene selection method based on the RF which yields small sets of genes [5]. This RF based gene selection method does not return sets of genes that are highly correlated because such genes would be redundant when it comes to predictive purposes. Since the RGLM based importance measure *timesSelectedByForwardRegression* is expected to lean towards selecting genes that are highly associated with the outcome, it comes as no surprise that only a few genes selected by the procedure of Díaz-Uriarte et al. turn out to have a top ranking in terms of the RGLM measure *timesSelectedByForwardRegression* (Additional file 5). It is beyond our scope to provide a thorough evaluation of the different variable selection approaches and we refer the reader to the literature, e.g. [5,64]. While our studies show that the RGLM based variable importance measures have some relationships to other measures, they are sufficiently different from other measures to warrant a thorough evaluation in future comparison studies.

#### RGLM predictor thinning based on a variable importance measure

Both RGLM and random forest have superior prediction accuracy but they differ with respect to how many features are being used. Recall that the random forest is composed of individual trees. Each tree is constructed by repeated node splits. The number of features considered at each node split is determined by the RF parameter *mtry*. The default value of *mtry* is the square root of the number of features. In case of 4999 gene features in our empirical studies, the default value is *mtry*=71. For RFbigmtry, we choose all possible features, i.e. *mtry*=4999. We find that a random forest predictor typically uses more than 40% of the features (i.e. more than 2000 genes) in the empirical studies. In contrast, RGLM typically only involves a few hundred genes in these studies. There are several reasons why RGLM uses far fewer features in its construction. First, and foremost, it uses forward selection (coupled with the AIC criterion) to select features in each bag. Second, the number of candidate covariates considered for forward regression is chosen to be low, i.e. *nCandidateCovariates*=50.

In RGLM, the number of times a feature is selected by forward regression models among all bags, *timesSelectedByForwardRegression*, follows a highly skewed distribution. Only few features are repeatedly selected into the model while most features are selected only once (if at all). It stands to reason that an even sparser, highly accurate predictor can be defined by refitting the GLM on each bag without considering these rarely selected features. We refer to this feature removal process as **RGLM predictor thinning**. Thus, features whose value of *timesSelectedByForwardRegression* lies below a pre-specified thinning threshold will be removed from the model fit a posteriori.

**Figure 9:**
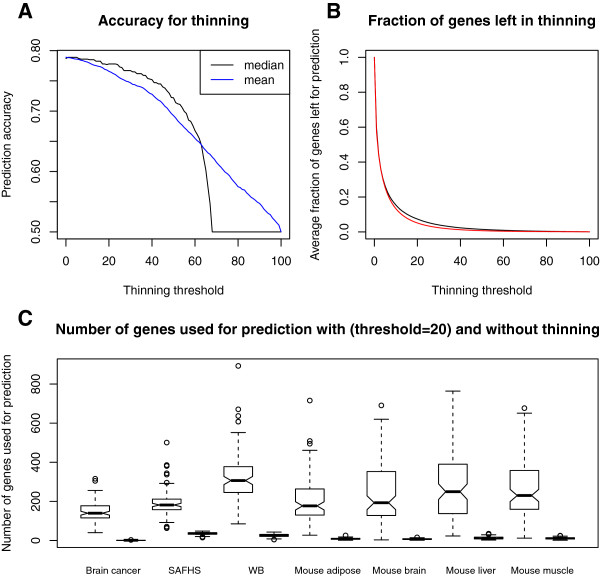
**RGLM predictor thinning.** This figure averages the thinning results of 700 applications (predicting 100 gene traits from each of 7 empirical data set). **(A)** Accuracies decrease as the thinning threshold increases. The black and blue lines represent the median and mean accuracies, respectively. **(B)** The average fraction of genes left in final models (y-axis) drops quickly as the thinning threshold increases as shown in the black line. The function in Equation 1 approximates the relationship between the two variables as shown in the red line. **(C)** Number of genes used in prediction for no thinning versus thinning threshold equal to 20. On average, less than 20% of genes remain.

Figure 9 presents the effects of predictor thinning in our empirical study. Here *nFeaturesInBag* is chosen to equal the total number of features. To ensure a fair comparison, we constructed and thinned the resulting RGLM in the training set only. Next, we evaluated the accuracy of the resulting thinned predictor in a test data set. Results were averaged across the 700 studies used in Figure 2 (A). Figure 9 (A) shows that the mean (and median) test set accuracies across 700 tests gradually decreases as the thinning threshold becomes more stringent. This is expected since the predictor loses potentially informative features with increasing values of the thinning threshold. Because the number of bags, *nBags*, is chosen to be 100, *timesSelectedByForwardRegression* takes on a value ≤100. Note that for a thinning threshold of 70 or larger, the median accuracy is constant at 0.5 which indicates that for at least 50% of comparisons the prediction is no longer informative. This reflects the fact that for large thinning thresholds, no covariates remain in the GLM models and the resulting predictor reduces to the “naive predictor” which assigns a constant outcome to all observations.

Interestingly, the accuracy diminishes very slowly for initial, low threshold values. But even low threshold values lead to a markedly sparser ensemble predictor (Figure 9 (B)). In other words, the average fraction of features (genes) remaining in the thinned RGLM declines drastically as the thinning threshold increases.

We have found that the following empirical function accurately describes the relationship between thinning threshold (*timesSelectedByForwardRegression* threshold) and proportion of features left in the thinned RGLM predictor:

(1) $$\;\begin{array}{l} \text{proportionLeft}=F(x)\\ \;=\left\{\begin{array}{l} 1\;\;\;\;\;\;\;\;\;x=0\\ \text{exp}\{-e{\left(\text{ex}\right)}^{0.775}\text{nBag}s^{0.0468(1-\text{log}(x))}\}\;0<x\leq 1 \end{array}\right) \end{array}$$

where $x=\frac{\text{thinning}\;\text{threshold}}{\text{nBags}}$ and *e* denotes Euler’s constant *e*≈2.718. Equation 1 was found by log transforming the data and using optimization approaches for estimating the parameters. No mathematical derivation was used. One can easily show that *F*(*x*) (Equation 1) is a monotonically decreasing function which accurately describes the proportion of remaining features as can be seen from Figure 9 (B). Since the proportion of remaining variables depends not only on the thinning threshold but also on the number of bags *nBags*, we also study how these results depend on the choice of *nBags*. Toward this end, we varied *nBags* from 20 to 500 for predicting the 100 dichotomized gene traits in the mouse adipose data set. Additional file 6 shows that the predicted values (red curve) based on Equation 1 overlaps almost perfectly with the observed values (black curve) for all considered choices of *nBags*, which indicates that Equation 1 accurately estimates the proportion of remaining features for range of different values of *nBags*.

Our results demonstrate that the number of required features decreases rapidly even for low values of the thinning threshold without compromising the prediction accuracy of the thinned predictor. Figure 9 (C) shows that a thinning threshold of 20, leads to a thinned predictor whose accuracy is negligibly lower (difference in median accuracy= 0.009) than that of the original RGLM predictor but it involves less than 20% of the original number of variables. Recall that even the original number of variables is markedly lower than that of the RF predictor. These results demonstrate that the thinned RGLM combines the advantages of an ensemble predictor (high accuracy) with that of a forward selected GLM model (few features, interpretability).

#### RGLM thinning versus RF thinning

**Figure 10:**
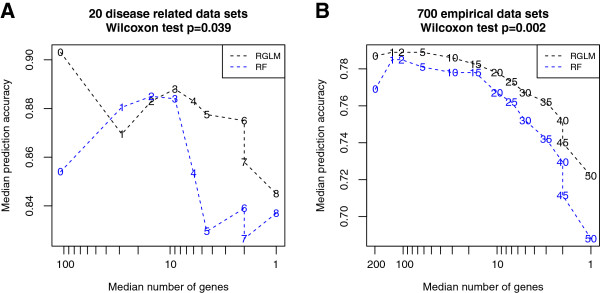
**RGLM thinning versus RF thinning.** This figure compares the thinned RGLM with the thinned RF in **(A)** the 20 disease related data sets and **(B)** the 700 gene expression traits. Numbers that connect dashed lines are RGLM thinning thresholds. For a pre-specified threshold, the number of features used for a thinned random forest is matched with that for the thinned RGLM (except for a threshold of 0). The *x*−*axis* (log-scaled) and the *y*−*axis* report the median number of genes left for prediction and the median accuracy across data sets, respectively. The Wilcoxon signed rank test was used to test whether the median accuracy of the thinned RGLM equals that of the thinned RF. Note that the thinned RGLM consistently yields higher accuracies than the thinned RF (according to the 2-sided test p-values).

The idea behind RGLM thinning is to remove features with low values of the variable importance measure. Of course, a similar idea can be applied to other predictors. Here we briefly evaluate the performance of a thinned random forest predictor which removed variables based on a low value of its importance measure (“mean decreased accuracy”). To arrive at an unbiased comparison, both RGLM and RF are thinned based on results obtained in the training data. Next, accuracies of the thinned predictors are evaluated in the test set data. Figure 10 compares thinned RGLM versus thinned RF in our disease related data sets and also the empirical studies. Numbers that connect dashed lines are RGLM thinning thresholds. For a pre-specified threshold, the number of features used in the thinned random forest is matched to that used in the thinned RGLM (except for the threshold 0). Without thinning, RF uses a lot more features than RGLM as mentioned previously. As expected, the median number of genes left for prediction and the corresponding median prediction accuracy generally decrease as the thinning threshold becomes more stringent. Overall, a thinned RGLM yields a significantly higher median accuracy than a thinned RF across different thinning thresholds (see the paired Wilcoxon signed rank test p-values). In clinical practice, a thinned predictor with very few features and good accuracy can be very useful and interpretable. For example, choosing a threshold of 5 in panel Figure 10 (A) and a threshold of 35 in panel (B) would result in very sparse predictors. In both cases, especially in panel (A), the thinned RGLM has higher median accuracy than that of the thinned RF.

## Discussion

### Why was the RGLM not discovered earlier?

**Figure 11:**
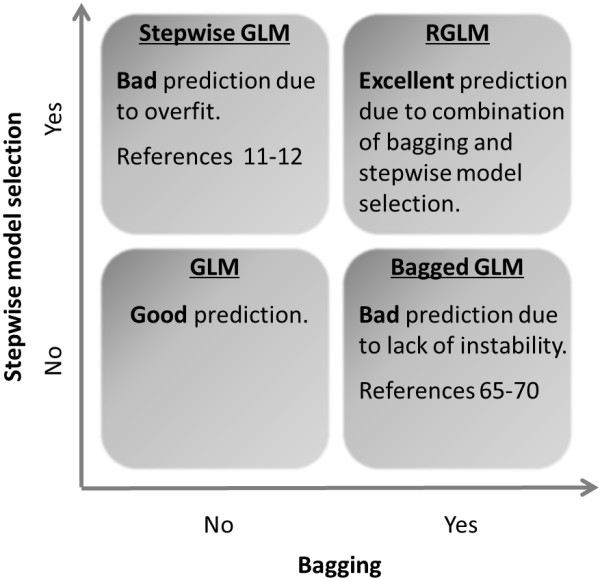
**How do modifications of a GLM affect the prediction accuracy.** The figure illustrates how two bad modifications to a GLM add up to a superior predictor (RGLM). In general, bagging or forward model selection alone lower the prediction accuracy of generalized linear models (such as logistic regression models). However, combining these two bad modifications leads to the superior prediction accuracy of the RGLM predictor. The figure may also explain why the benefits of RGLM type predictors were not previously recognized.

After Breiman proposed the idea of bagged linear regression models in 1996 [10], many authors have explored the utility of bagging logistic regression models [65-71]. Most previous studies report that bagging does not improve the accuracy of logistic regression. Bühlman and Yu showed theoretically that bagging helps for “hard threshold” methods but not for “soft threshold” methods (such as logistic regression) [72]. These studies indicate that bagged logistic regression models are not beneficial since the individual predictors (logistic regression models) are too stable. Overall, we agree with these results. But our comprehensive evaluations show that by injecting elements of randomness and instability into a bagged logistic regression model one arrives at a state of the art prediction method that often outperforms existing methods. Figure 11 describes why the construction of the RGLM runs counter to conventional wisdom. As indicated by the upper right hand panel of Figure 11, the RGLM is based on two seemingly bad modifications to a GLM. As indicated by the top left panel of Figure 11, forward selection of a GLM is typically a bad idea since it overfits the data and thus degrades the prediction accuracy of a single GLM predictor. As indicated by the bottom right panel of Figure 11, bagging a full logistic regression (i.e. without variable selection) is also a bad idea since it leads to a complicated (ensemble) predictor without clear evidence for increased accuracy (see related articles by [65-70]). But these two seemingly bad modifications add up to a superior prediction method (i.e. minus times minus equals plus). Breiman already noted that the instability afforded by variable selection is important for constructing a bagged linear model based predictor [10]. In order to define an accurate GLM based ensemble predictor, we also find that it is important to introduce additional elements of randomness and instability, which is also reflected in the name *random* GLM. Our results show that the proposed changes (allowing for interaction terms, forward variable selection using AIC, restricting the number of features per bag and the number of candidate features) results in a more accurate predictor that involves surprisingly few features (especially when thinning is used).

Additional reasons why the merits of RGLM have not been recognized earlier may be the following. First, it may be a historical accident. Bagging was quickly over-shadowed by other seemingly more accurate ways of constructing ensemble predictors, such as boosting [73] and the RF [13], both of which have markedly better performance on the UCI benchmark data. We find that RGLM.inter2 ties with SVM and RF for the top spot in UCI benchmark data set (Table 5). Incidentally, RGLM performs significantly better than SVM and RF on the disease data sets (Table 4) and in the 700 gene expression comparisons (Figure 2).

Second, previous comparisons of bagged predictors in the context of genomic data were based on limited empirical evaluations. Many comparisons involved fewer than 20 microarray data sets when comparing predictors [4,5]. While the comparisons involved clinically important data sets from cancer applications, these studies were simply not comprehensive enough.

Third, previous studies probably did not consider enough bootstrap samples (bags). While previous studies used 10 to 50 bags, we always used 100 bags when constructing the RGLM. To illustrate how prediction accuracy depends on the number of bags, we evaluate the brain cancer data with 1 to 500 bags using 5 gene traits randomly selected from those used in our binary and continuous outcome prediction, respectively. The results are shown in Additional file 7. Most improvement is gained in the first several dozens of bags. 100 bags is generally enough although fluctuations remain. More bags may lead to slightly better predictions but at the expense of longer computation time.

### Strengths and limitations

RGLM shares many advantages of bagged predictors including a nearly unbiased estimate of the prediction accuracy (the out-of-bag estimate) and several variable importance measures. While our empirical studies focus on binary and continuous outcomes, it is straightforward to define RGLM for count outcomes (resulting in a random Poisson regression model) and for multi-class outcomes (resulting in a random multinomial regression model).

A noteworthy limitation of RGLM is computational complexity since the forward selection process (e.g. by the function *stepAIC*[22] from the *MASS* R package) is particularly time-consuming. The total time depends on the number of candidate features, the order of interaction terms, and the number of bags. Our R implementation allows the user to use parallel processing for speeding up the calculations.

Our empirical studies demonstrate that RGLM compares favorably with the random forest, support vector machines, penalized regression models, and many other widely used prediction methods. As a caveat, we mention that we chose default parameter choices for each of these methods in order to ensure a fair comparison. Future studies could evaluate how these prediction methods compare when resampling schemes (e.g. cross validation) are used to inform parameter choices. Our *randomGLM* R package will allow the reader to carefully evaluate the method.

## Conclusions

Since individual forward selected GLMs are highly interpretable, the resulting ensemble predictor is more interpretable than an RF predictor. Our empirical studies (20 disease related gene expression data sets, 700 gene expression trait data, the UCI benchmark data) clearly highlight the outstanding prediction accuracy afforded by the RGLM. High accuracies are achieved not only in genomic data sets (many features, small sample size) but also in the UCI benchmark data (few features, large sample size).

## Abbreviations

RGLM: Random generalized linear model; RGLM: Inter2 - RGLM considering pairwise interactions between features; RGLM: Inter3 - RGLM considering two-way and three-way interactions between features; forwardGLM: Forward selected generalized linear model; RF: Random forest with default mtry; RFbigmtry: Random forest with mtry equal to the total number of features; GLM: Generalized linear model; Rpart: Recursive partitioning; LDA: Linear discriminant analysis; DLDA: Diagonal linear discriminant analysis; KNN: K nearest neighbor; SVM: Support vector machine; SC: Shrunken centroids; RSM: Random subspace method; RMNL: Random multinomial logit model; RKNN: Random K nearest neighbor; E-RFE: Entropy-based recursive feature elimination; AIC: Akaike information criteria; aMV: Adjusted majority vote.

## Competing interests

The authors declare that they have no competing interest.

## Authors’ contributions

LS carried out all analyses. PL helped with the R implementation and analysis. LS and SH developed the method and wrote the article. SH conceived of the study. All authors read and approved the final manuscript.

## Supplementary Material

Additional file 1 **Simulation study design.** This file describes the simulation studies and presents R code used for simulating the data set. Click here for file

Additional file 2 **Sensitivity and specificity of predictors in the 20 disease gene expression data sets.** For each data set and prediction method, the table reports the sensitivity and specificity estimated using 3-fold cross validation. More precisely, the table reports the average 3-fold CV estimate over 100 random partitions of the data into 3 folds. Median sensitivity and specificity across data sets are summarized at the bottom. Click here for file

Additional file 3 **Sensitivity and specificity of predictors in the UCI machine learning benchmark data.** For each data set and prediction method, the table reports the sensitivity and specificity estimated using 3-fold cross validation. More precisely, the table reports the average 3-fold CV estimate over 100 random partitions of the data into 3 folds. Median sensitivity and specificity across data sets are summarized at the bottom. Click here for file

Additional file 4 **Prediction accuracy when including pairwise interactions between features in the UCI machine learning benchmark data.** This table is an extension to Table 5. It shows the prediction accuracy of predictors other than RGLM when considering pairwise interactions between features in the same UCI mlbench data sets. Although several predictors show improvement, none of them beats RGLM.inter2. Click here for file

Additional file 5 **Comparison of RGLM based feature selection method with the RF based method of Díaz-Uriarte et al.** For each data set in the 20 disease gene expression data, the RF based variable selection method by Díaz-Uriarte *et al* selects a small set of genes. For each of the selected genes, the file reports the ranking in terms of the RGLM variable importance measure *timesSelectedByForwardRegression*. As expected, only a few of the selected genes have a high rank in terms of *timesSelectedByForwardRegression* illustrating that these variable selection methods are different. Click here for file

Additional file 6 **Effect of the number of bags on RGLM predictor thinning.** s This figure reports how prediction accuracy changes as variable thinning is applied to the RGLM. Results are averaged over the 100 dichotomized gene traits in the mouse adipose data set. The five rows correspond to *nBags* values of 20, 50, 100, 200, 500 respectively. Within each row, the two panels have the same meaning as in Figure 9. Click here for file

Additional file 7 **Prediction accuracy versus number of bags used for RGLM.** This figure presents the results for predicting 5 gene traits in the brain cancer data set when different numbers of bags (bootstrap samples) are used for constructing the RGLM. Each color represents one gene trait. **(A)** Binary outcome prediction. The 5 gene traits were randomly selected from all 100 gene traits used in the binary outcome prediction section. **(B)** Continuous outcome prediction. The 5 gene traits were randomly selected from all 100 gene traits used in the continuous outcome prediction. Click here for file
