# Supplementary material for: Random generalized linear model: a highly accurate and interpretable ensemble predictor
Source: BMC Bioinformatics. 2013 Jan 16;14:5. doi: 10.1186/1471-2105-14-5 (PMC3645958; doi:10.1186/1471-2105-14-5)
Supplement: Additional file 1 — Simulation study design. This file describes the simulation studies and presents R code used for simulating the data set. [file 1471-2105-14-5-S1.pdf]

The simulation design is described in (Horvath 2011. Springer Book, section 6.13) but we briefly summarize the main points. The gene expression data are stored in a m×n dimensional numeric matrix datExpr. The n columns of datExpr correspond to gene expression profiles that follow a normal distribution across m samples. We assume five modules (clusters) underlying the expression data. Clusters are color coded (and labeled) by turquoise, blue, brown, green, and yellow. Only two of these modules (brown and green) are simulated to relate to y. Not all genes are simulated to be part of a module: a relatively large number of genes will be simulated outside of the proper modules and will be color-coded gray. A co-expression module is simulated to be a matrix whose columns correspond to genes with varying correlations its seed module eigengene. Thus, the resulting module genes have different levels of correlations with the seed module eigengene. The seed module eigengene is not part of the module.

The simulation proceeds along two steps. First, seed module eigengenes are simulated, so that the brown and green eigengenes are correlated with the sample trait y with correlation -0.6 and 0.6. Second, gene expression vectors are simulated around the seed module eigengenes. Toward this end, we use the function simulateDatExpr5Modules. Module sizes (proportions) are specified using the function argument simulateProportions. Proportions in the turquoise, blue, brown, yellow, green modules are 0.2, 0.15, 0.08, 0.06 and 0.04, respectively. Note that the proportions don't add up to 1 since we allow for extra-modular background genes, colored in gray.

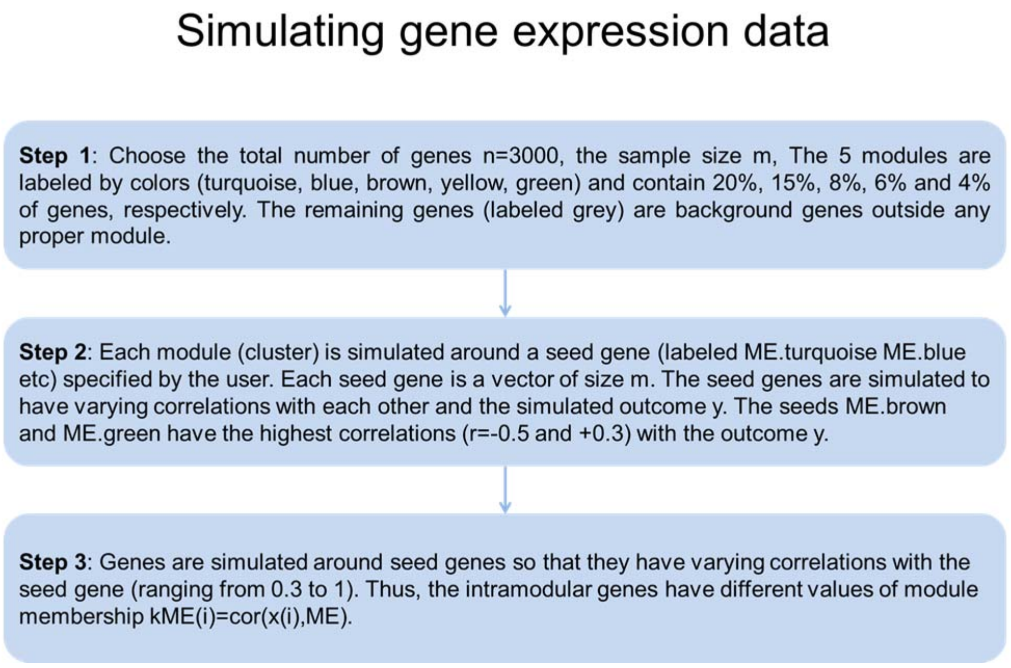

Figure 1. Visualization of simulation design.

The simulation design is visualized in Figure 1. The following is the R function used in this article to simulate one data sets given the number of samples in training set, the number of samples in test set, the number of genes and random seed.

```
simulationTrainTest = function(nSamplesTrain, nSamplesTest, nGenes, seed)
{
  set.seed(seed)
  # now we specify the true measures of eigengene significance
  # recall that ESturquoise=cor(y,MEturquoise)
  ESturquoise=0;
  ESbrown= -.6;
  ESgreen=.6;
  ESyellow=0
  # Note that we dont specify the eigengene significance of the blue module
  # since it is highly correlated with the turquoise module.
  ESvector=c(ESturquoise,ESbrown,ESgreen,ESyellow)

  # proportion of genes in the turquoise, blue, brown, green, and yellow module respectively.
  simulateProportions1=c(0.2,0.15, 0.08, 0.06, 0.04)

### simulate train set
  #Step 1: simulate a module eigengene network.
  # Training Data Set I
  MEgreen=rnorm(nSamplesTrain)
  scaledy=MEgreen*ESgreen+sqrt(1-ESgreen^2)*rnorm(nSamplesTrain)
  y1=ifelse( scaledy>median(scaledy), 1,0)
  MEturquoise= ESturquoise*scaledy+sqrt(1-ESturquoise^2)*rnorm(nSamplesTrain)
  # we simulate a strong dependence between MEblue and MEturquoise
  MEblue= .6*MEturquoise+ sqrt(1-.6^2) *rnorm(nSamplesTrain)
  MEbrown= ESbrown*scaledy+sqrt(1-ESbrown^2)*rnorm(nSamplesTrain)
  MEyellow= ESyellow*scaledy+sqrt(1-ESyellow^2)*rnorm(nSamplesTrain)

ModuleEigengeneNetwork1=data.frame(y1,MEturquoise,MEblue,MEbrown,MEgreen, MEyellow)

  # Step 2: Simulating gene expressions around the module eigengenes
  dat1=simulateDatExpr5Modules(
      MEturquoise=ModuleEigengeneNetwork1$MEturquoise,
      MEblue=ModuleEigengeneNetwork1$MEblue,
      MEbrown=ModuleEigengeneNetwork1$MEbrown,
      MEyellow=ModuleEigengeneNetwork1$MEyellow,
      MEgreen=ModuleEigengeneNetwork1$MEgreen,
      nGenes=nGenes,
      simulateProportions=simulateProportions1)
  datExpr = dat1$datExpr;
  dimnames(datExpr)[[1]]=paste("Sample",1:dim(datExpr)[[1]], sep="" )
  dimnames(datExpr)[[2]]=paste("Gene",1:dim(datExpr)[[2]], sep="" )

  rm(dat1); collectGarbage();
```

```
### simulate test set

  set.seed(seed+1)
  MEgreen=rnorm(nSamplesTest)
  scaledy2=MEgreen*ESgreen+sqrt(1-ESgreen^2)*rnorm(nSamplesTest)
  y2=ifelse( scaledy2>median(scaledy2),1,0)
  MEturquoise=                              ESturquoise*scaledy2+sqrt(1-
ESturquoise^2)*rnorm(nSamplesTest)
  # we simulate a strong dependence between MEblue and MEturquoise
  MEblue= .6*MEturquoise+ sqrt(1-.6^2) *rnorm(nSamplesTest)
  MEbrown= ESbrown*scaledy2+sqrt(1-ESbrown^2)*rnorm(nSamplesTest)
  MEyellow= ESyellow*scaledy2+sqrt(1-ESyellow^2)*rnorm(nSamplesTest)
  # Put together a data frame of eigengenes

ModuleEigengeneNetwork2=data.frame(y=y2,MEturquoise,MEblue,MEbrown,MEgr
een, MEyellow)
  # Simulate the expression data
  dat2=simulateDatExpr5Modules(
      MEturquoise=ModuleEigengeneNetwork2$MEturquoise,
      MEblue=ModuleEigengeneNetwork2$MEblue,
      MEbrown=ModuleEigengeneNetwork2$MEbrown,
      MEyellow=ModuleEigengeneNetwork2$MEyellow,
      MEgreen=ModuleEigengeneNetwork2$MEgreen,
      simulateProportions=simulateProportions1,
      nGenes=nGenes)
  datExprTest = dat2$datExpr

  dimnames(datExprTest)[[1]]=paste("SampleTest",1:dim(datExprTest)[[1]],
sep="" )
  dimnames(datExprTest)[[2]]=paste("Gene",1:dim(datExprTest)[[2]],
sep="" )

  out = list(dat1=datExpr, y1=y1, dat2=datExprTest, y2=y2)
  out
}
```

Reference

Horvath S: Weighted Network Analysis. Applications in Genomics and Systems Biology. Springer Book 2011.
