# Supplementary material for: Random generalized linear model: a highly accurate and interpretable ensemble predictor
Source: BMC Bioinformatics. 2013 Jan 16;14:5. doi: 10.1186/1471-2105-14-5 (PMC3645958; doi:10.1186/1471-2105-14-5)
Supplement: Additional file 2 — Sensitivity and specificity of predictors in the 20 disease gene expression data sets. For each data set and prediction method, the table reports the sensitivity and specificity estimated using 3-fold cross validation. More precisely, the table reports the average 3-fold CV estimate over 100 random partitions of the data into 3 folds. Median sensitivity and specificity across data sets are summarized at the bottom. [file 1471-2105-14-5-S2.pdf]

Sensitivity

Data set	RGLM	RF	RFbigmtry	Rpart	LDA	DLDA	KNN	SVM	SC
adenocarcinoma	0.984	0.984	0.984	0.828	0.953	0.813	0.984	1.000	0.938
brain	0.969	0.969	0.969	0.813	1.000	0.938	0.891	1.000	0.969
breast2	0.485	0.424	0.455	0.515	0.455	0.515	0.333	0.273	0.470
breast3	0.706	0.706	0.725	0.627	0.706	0.588	0.627	0.686	0.627
colon	0.773	0.682	0.727	0.636	0.818	0.864	0.591	0.545	0.864
leukemia	1.000	1.000	1.000	0.889	0.963	0.963	1.000	1.000	0.963
lymphoma	1.000	1.000	1.000	0.850	0.950	1.000	1.000	1.000	1.000
NCI60	1.000	1.000	1.000	0.808	1.000	0.981	0.942	1.000	1.000
prostate	0.960	0.920	0.900	0.840	0.880	0.560	0.800	0.880	0.940
srbct	1.000	1.000	1.000	0.950	0.950	0.950	0.975	1.000	1.000
BrainTumor2	0.636	0.591	0.636	0.591	0.682	0.727	0.727	0.409	0.773
DLBCL	0.966	0.983	0.966	0.879	0.983	0.810	0.862	1.000	0.845
lung1	0.833	0.778	0.778	0.722	0.722	0.833	0.889	0.667	0.889
lung2	0.938	0.969	0.969	0.875	0.969	0.969	0.969	0.969	0.969
lung3	0.868	0.868	0.868	0.789	0.895	0.816	0.842	0.868	0.829
psoriasis1	0.984	0.992	0.992	0.992	0.992	0.992	0.992	0.992	0.992
psoriasis2	0.980	1.000	1.000	0.980	0.980	0.980	0.980	1.000	0.980
MSstage1	0.929	0.929	0.929	0.571	0.929	1.000	0.857	0.929	0.929
MSdiagnosis1	1.000	1.000	1.000	1.000	1.000	1.000	1.000	1.000	1.000
MSdiagnosis2	0.389	0.389	0.444	0.500	0.333	0.778	0.222	0.111	0.472
MedianSensitivity	0.960	0.969	0.950	0.813	0.938	0.889	0.904	0.969	0.938

Specificity

Data set	RGLM	RF	RFbigmtry	Rpart	LDA	DLDA	KNN	SVM	SC
adenocarcinoma	0.167	0.083	0.042	0.250	0.250	0.375	0.083	0.000	0.083
brain	0.500	0.300	0.400	0.600	0.200	0.900	0.900	0.100	0.900
breast2	0.705	0.750	0.750	0.625	0.727	0.727	0.773	0.773	0.750
breast3	0.693	0.705	0.705	0.614	0.682	0.841	0.727	0.648	0.773
colon	0.875	0.900	0.900	0.775	0.875	0.850	0.875	0.925	0.875
leukemia	0.727	0.636	0.727	0.682	0.636	1.000	1.000	0.182	1.000
lymphoma	0.976	1.000	1.000	0.929	0.976	0.976	0.976	1.000	0.976
NCI60	0.333	0.111	0.222	0.333	0.222	0.556	0.333	0.111	0.444
prostate	0.904	0.865	0.904	0.865	0.865	0.692	0.817	0.827	0.904
srbct	1.000	0.870	0.957	0.870	0.696	0.826	0.913	0.652	1.000
BrainTumor2	0.857	0.857	0.821	0.643	0.821	0.679	0.714	0.821	0.714
DLBCL	0.737	0.526	0.632	0.684	0.737	0.632	0.895	0.158	0.921
lung1	1.000	1.000	1.000	0.900	1.000	0.975	0.975	1.000	0.925
lung2	0.929	0.857	0.857	0.714	0.929	1.000	0.857	0.571	1.000
lung3	0.939	0.909	0.909	0.788	0.879	0.909	0.833	0.818	0.939
psoriasis1	0.983	1.000	0.983	0.966	1.000	0.983	0.983	0.966	0.983
psoriasis2	0.939	0.970	0.970	0.939	0.970	0.939	0.970	0.909	0.939
MSstage1	0.833	0.833	0.833	0.167	0.667	0.583	0.750	0.583	0.583
MSdiagnosis1	0.917	0.833	0.833	0.000	0.750	0.750	0.917	0.833	0.833
MSdiagnosis2	0.731	0.808	0.769	0.654	0.692	0.462	0.808	0.885	0.538
MedianSpecificity	0.857	0.833	0.833	0.702	0.750	0.825	0.848	0.750	0.900
