# Supplementary material for: Random generalized linear model: a highly accurate and interpretable ensemble predictor
Source: BMC Bioinformatics. 2013 Jan 16;14:5. doi: 10.1186/1471-2105-14-5 (PMC3645958; doi:10.1186/1471-2105-14-5)
Supplement: Additional file 3 — Sensitivity and specificity of predictors in the UCI machine learning benchmark data. For each data set and prediction method, the table reports the sensitivity and specificity estimated using 3-fold cross validation. More precisely, the table reports the average 3-fold CV estimate over 100 random partitions of the data into 3 folds. Median sensitivity and specificity across data sets are summarized at the bottom. [file 1471-2105-14-5-S3.pdf]

Sensitivity

Data set	RGLM	RGLM.inter2	RF	RFbigmtry	Rpart	LDA	DLDA	KNN	SVM	SC
**BreastCancer**	0.974	0.967	0.972	0.969	0.948	0.978	0.974	0.974	0.965	0.976
**HouseVotes84**	0.962	0.962	0.959	0.955	0.951	0.932	0.891	0.914	0.959	0.944
**Ionosphere**	0.746	0.889	0.873	0.865	0.786	0.651	0.738	0.623	0.865	0.627
**diabetes**	0.876	0.855	0.852	0.838	0.824	0.878	0.746	0.850	0.870	0.914
**Sonar**	0.802	0.910	0.883	0.838	0.743	0.748	0.667	0.865	0.874	0.784
**ringnorm**	0.453	0.973	0.940	0.900	0.767	0.470	0.473	0.193	0.953	0.477
**threenorm**	0.827	0.833	0.827	0.787	0.653	0.840	0.853	0.707	0.850	0.847
**twonorm**	0.933	0.940	0.940	0.913	0.740	0.940	0.933	0.913	0.933	0.933
**Glass**	0.841	0.826	0.913	0.877	0.815	0.851	0.457	0.891	0.884	1.000
**Satellite**	0.992	0.990	0.993	0.993	0.975	0.994	0.723	0.994	0.991	0.896
**Vehicle**	0.976	0.994	0.994	0.981	0.959	0.979	0.744	0.938	0.989	0.994
**Vowel**	0.977	0.993	1.000	0.993	0.976	0.968	0.857	1.000	0.998	1.000
**MedianSensitivity**	0.892	0.955	0.940	0.907	0.826	0.906	0.746	0.902	0.947	0.927

Specificity

Data set	RGLM	RGLM.inter2	RF	RFbigmtry	Rpart	LDA	DLDA	KNN	SVM	SC
**BreastCancer**	0.946	0.946	0.963	0.950	0.929	0.917	0.929	0.950	0.971	0.917
**HouseVotes84**	0.958	0.964	0.958	0.952	0.961	0.982	0.952	0.934	0.958	0.931
**Ionosphere**	0.964	0.978	0.964	0.947	0.924	0.978	0.849	0.978	0.982	0.942
**diabetes**	0.567	0.584	0.586	0.597	0.588	0.562	0.701	0.534	0.541	0.429
**Sonar**	0.722	0.753	0.742	0.722	0.649	0.701	0.732	0.753	0.763	0.660
**ringnorm**	0.700	0.973	0.940	0.923	0.780	0.660	0.670	0.987	0.993	0.597
**threenorm**	0.793	0.820	0.787	0.773	0.647	0.793	0.793	0.920	0.860	0.787
**twonorm**	0.940	0.967	0.960	0.927	0.740	0.977	0.987	0.980	0.973	0.980
**Glass**	0.257	0.592	0.658	0.658	0.553	0.316	0.671	0.658	0.513	0.000
**Satellite**	0.967	0.978	0.969	0.962	0.914	0.955	0.770	0.978	0.975	0.510
**Vehicle**	0.931	0.968	0.963	0.952	0.899	0.931	0.688	0.828	0.931	0.064
**Vowel**	0.533	0.900	0.822	0.800	0.700	0.656	0.800	1.000	0.933	0.000
**MedianSpecificity**	0.873	0.954	0.947	0.926	0.767	0.881	0.771	0.946	0.952	0.639
