# Supplementary material for: Random generalized linear model: a highly accurate and interpretable ensemble predictor
Source: BMC Bioinformatics. 2013 Jan 16;14:5. doi: 10.1186/1471-2105-14-5 (PMC3645958; doi:10.1186/1471-2105-14-5)
Supplement: Additional file 4 — Prediction accuracy when including pairwise interactions between features in the UCI machine learning benchmark data. This table is an extension to Table 5. It shows the prediction accuracy of predictors other than RGLM when considering pairwise interactions between features in the same UCI mlbench data sets. Although several predictors show improvement, none of them beats RGLM.inter2. [file 1471-2105-14-5-S4.pdf]

Data set	RF	RFbigmtry	Rpart	LDA	DLDA	KNN	SVM	SC
**BreastCancer**	0.969	0.964	0.954	0.963	0.940	0.963	0.969	0.949
**HouseVotes84**	0.958	0.951	0.944	0.903	0.914	0.924	0.961	0.944
**Ionosphere**	0.932	0.923	0.880	0.707	0.764	0.832	0.929	0.852
**diabetes**	0.757	0.750	0.732	0.760	0.736	0.728	0.755	0.751
**Sonar**	0.812	0.830	0.760	0.755	0.686	0.812	0.812	0.707
**ringnorm**	0.953	0.922	0.760	0.753	0.873	0.557	0.970	0.943
**threenorm**	0.777	0.753	0.612	0.620	0.853	0.665	0.807	0.847
**twonorm**	0.937	0.897	0.727	0.717	0.933	0.732	0.923	0.953
**Glass**	0.813	0.790	0.732	0.710	0.559	0.788	0.734	0.617
**Satellite**	0.988	0.986	0.962	0.988	0.708	0.990	0.988	0.762
**Vehicle**	0.984	0.978	0.946	0.984	0.771	0.892	0.969	0.779
**Vowel**	0.991	0.986	0.949	0.982	0.899	1.000	0.997	0.912
**MedianAccuracy**	0.947	0.926	0.835	0.764	0.802	0.829	0.946	0.849
