# Supplementary material for: Random generalized linear model: a highly accurate and interpretable ensemble predictor
Source: BMC Bioinformatics. 2013 Jan 16;14:5. doi: 10.1186/1471-2105-14-5 (PMC3645958; doi:10.1186/1471-2105-14-5)
Supplement: Additional file 5 — Comparison of RGLM based feature selection method with the RF based method of Díaz-Uriarte et al. For each data set in the 20 disease gene expression data, the RF based variable selection method by Díaz-Uriarte et al selects a small set of genes. For each of the selected genes, the file reports the ranking in terms of the RGLM variable importance measure timesSelectedByForwardRegression. As expected, only a few of the selected genes have a high rank in terms of timesSelectedByForwardRegression illustrating that these variable selection methods are different. [file 1471-2105-14-5-S5.pdf]

| Data set | Ranks |
|---|---|
| adenocarcinoma | 1 17 17 259 259 259 |
| brain | 1 31 |
| breast2 | 1 4 16 16 16 120 406 406 406 |
| breast3 | 2 8 13 26 40 40 180 |
| colon | 1 2 5 |
| leukemia | 2 17 |
| lymphoma | 2 23 |
| NCI60 | 1 13 25 144 |
| prostate | 1 2 |
| srbct | 1 2 4 |
| BrainTumor2 | 1 3 21 56 237 237 |
| DLBCL | 6 163 |
| lung1 | 1 2 2 4 11 11 19 19 19 38 115 115 |
| lung2 | 19 19 |
| lung3 | 3 19 |
| psoriasis1 | 33 33 |
| psoriasis2 | 3 63 |
| MSstage1 | 1 3 3 8 8 27 101 101 101 101 101 101 101 101 101 |
| MSdiagnosis1 | 57 57 |
| MSdiagnosis2 | 261 261 261 |
