# Supplementary figures and images for: Random generalized linear model: a highly accurate and interpretable ensemble predictor
Source: BMC Bioinformatics. 2013 Jan 16;14:5. doi: 10.1186/1471-2105-14-5 (PMC3645958; doi:10.1186/1471-2105-14-5)

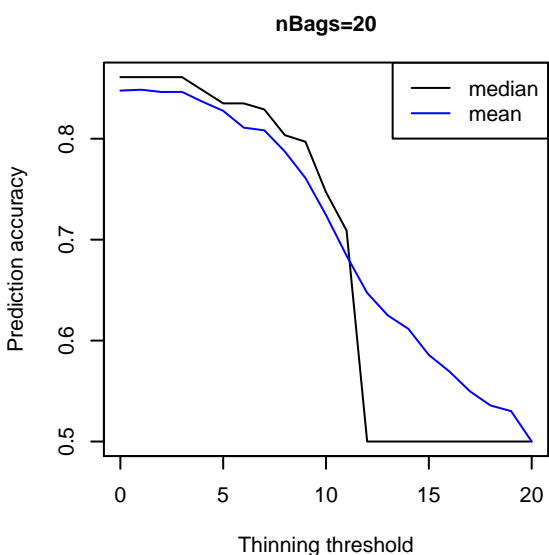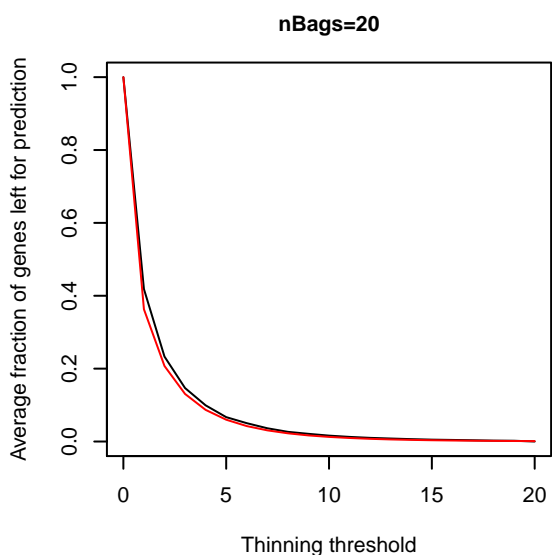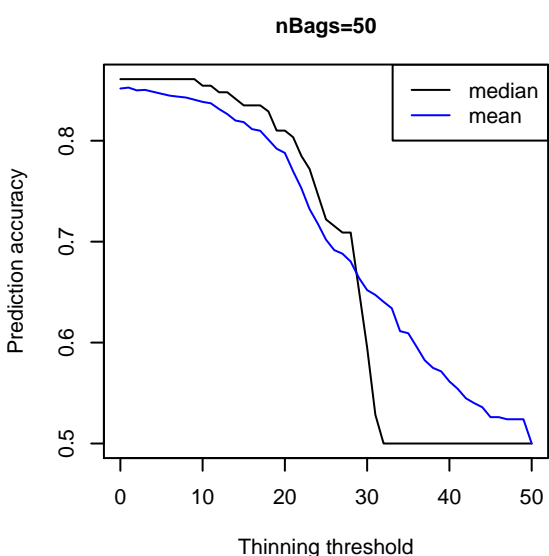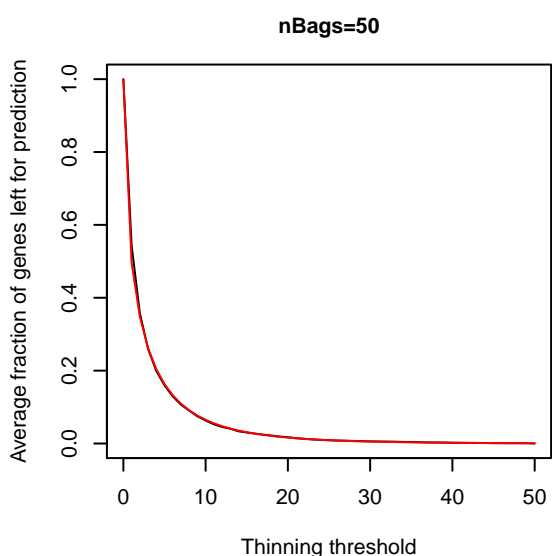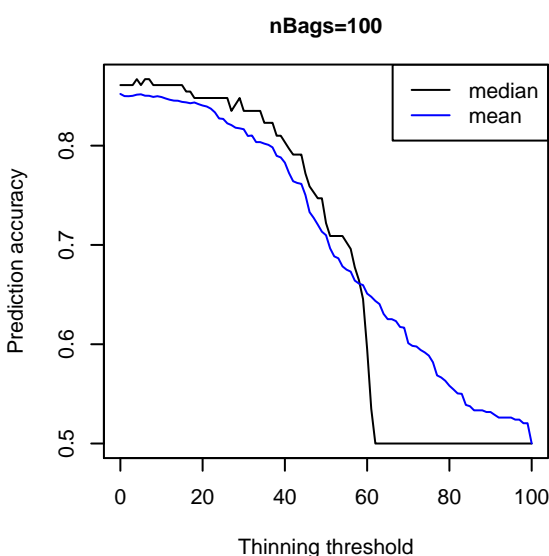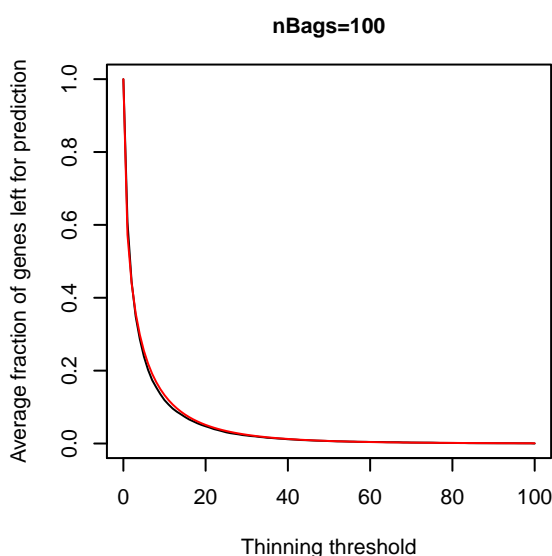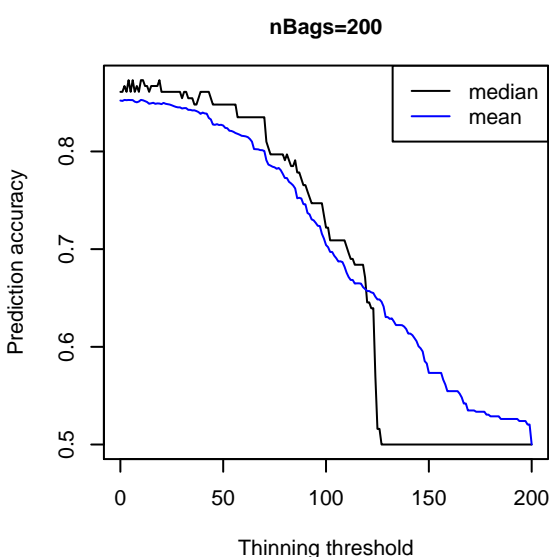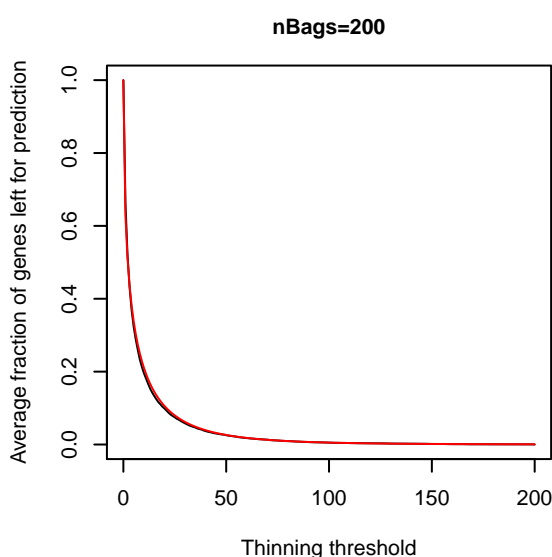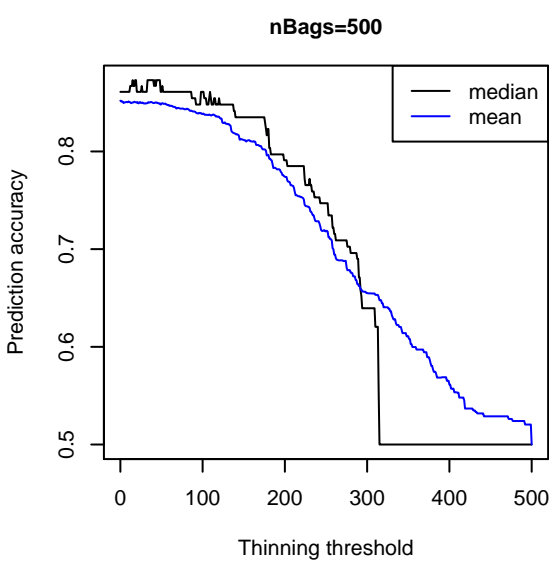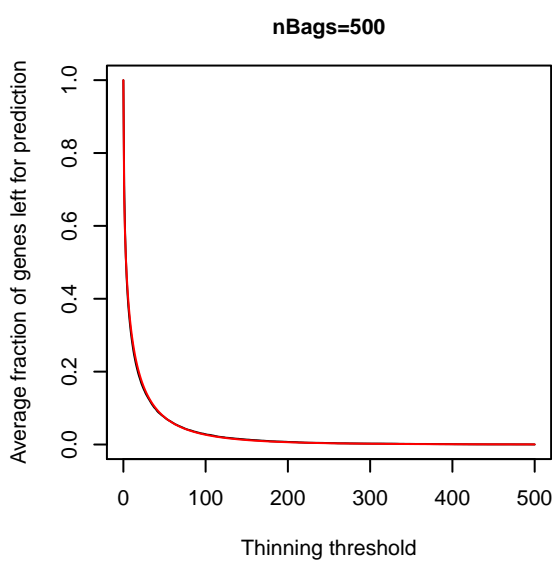

Supplement: Additional file 6 — Effect of the number of bags on RGLM predictor thinning. s This figure reports how prediction accuracy changes as variable thinning is applied to the RGLM. Results are averaged over the 100 dichotomized gene traits in the mouse adipose data set. The five rows correspond to nBags values of 20, 50, 100, 200, 500 respectively. Within each row, the two panels have the same meaning as in Figure 9. [file 1471-2105-14-5-S6.pdf]

**A****Binary outcome, 5 randomly selected traits**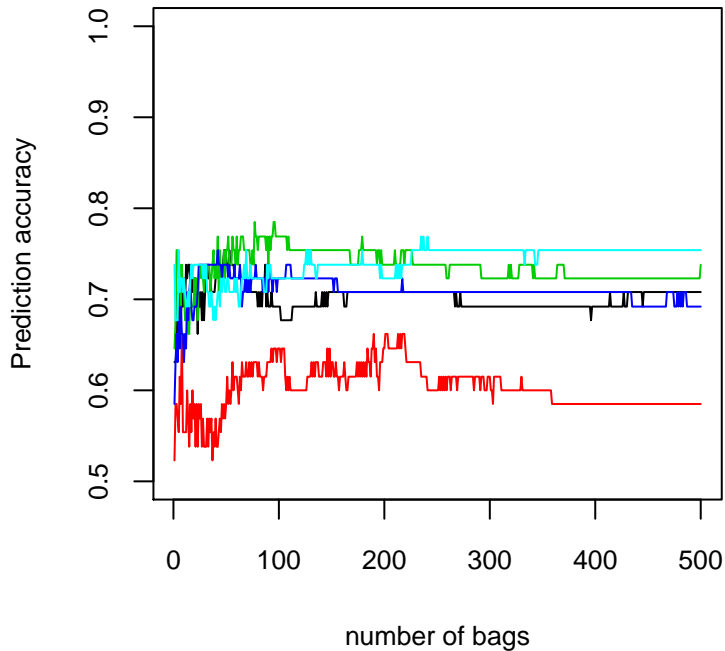**B****Continuous outcome, 5 randomly selected traits**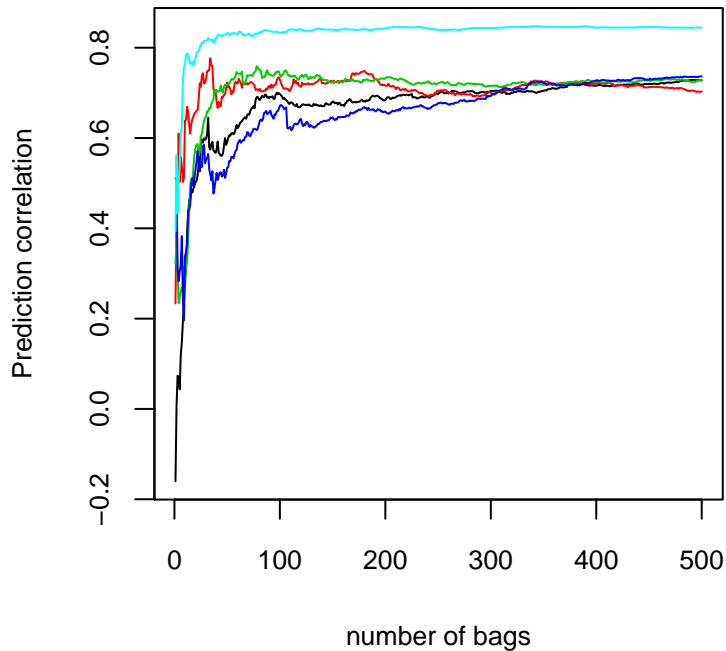

Supplement: Additional file 7 — Prediction accuracy versus number of bags used for RGLM. This figure presents the results for predicting 5 gene traits in the brain cancer data set when different numbers of bags (bootstrap samples) are used for constructing the RGLM. Each color represents one gene trait. (A) Binary outcome prediction. The 5 gene traits were randomly selected from all 100 gene traits used in the binary outcome prediction section. (B) Continuous outcome prediction. The 5 gene traits were randomly selected from all 100 gene traits used in the continuous outcome prediction. [file 1471-2105-14-5-S7.pdf]
